# Locally Addressable Energy Efficient Actuation of Magnetic Soft Actuator Array Systems

**DOI:** 10.1002/advs.202302077

**Published:** 2023-06-17

**Authors:** Michiel Richter, Jakub Sikorski, Pavlo Makushko, Yevhen Zabila, Venkatasubramanian Kalpathy Venkiteswaran, Denys Makarov, Sarthak Misra

**Affiliations:** ^1^ Surgical Robotics Laboratory Department of Biomechanical Engineering University of Twente Drienerlolaan 5 Enschede 7500 AE The Netherlands; ^2^ Surgical Robotics Laboratory Department of Biomedical Engineering University of Groningen and University Medical Centre Groningen, Hanzeplein 1 Groningen 9713 GZ The Netherlands; ^3^ Institute of Ion Beam Physics and Materials Research, Helmholtz‐Zentrum Dresden‐Rossendorf e.V. Bautzner, Landstraße 400 01328 Dresden Germany; ^4^ The H. Niewodniczanski Institute of Nuclear Physics, Polish Academy of Sciences Krakow 31‐342 Poland

**Keywords:** independent actuation, magnetic near‐field, magnetic soft machines, planar coils

## Abstract

Advances in magnetoresponsive composites and (electro‐)magnetic actuators have led to development of magnetic soft machines (MSMs) as building blocks for small‐scale robotic devices. Near‐field MSMs offer energy efficiency and compactness by bringing the field source and effectors in close proximity. Current challenges of near‐field MSM are limited programmability of effector motion, dimensionality, ability to perform collaborative tasks, and structural flexibility. Herein, a new class of near‐field MSMs is demonstrated that combines microscale thickness flexible planar coils with magnetoresponsive polymer effectors. Ultrathin manufacturing and magnetic programming of effectors is used to tailor their response to the nonhomogeneous near‐field distribution on the coil surface. The MSMs are demonstrated to lift, tilt, pull, or grasp in close proximity to each other. These ultrathin (80 µm) and lightweight (100 gm^−2^) MSMs can operate at high frequency (25 Hz) and low energy consumption (0.5 W), required for the use of MSMs in portable electronics.

## Graphical abstract

Magnetic soft machines (MSMs) are composed of flexible planar electromagnetic coils and magnetic polymer composite effectors positioned directly on the coil surface. The magnetic near‐field of the coils combined with non‐uniform magnetization profiles on the effectors permit development of adjacent and independently actuated MSMs able to lift, tilt, pull, or grab in close proximity to each other.

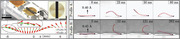

## 1 Introduction

The use of soft materials in mechatronics has opened up a vast design space permitting the development of new classes of machines.^[^ ^1^, ^2^, ^3^, ^4^, ^5^ ^]^ Related developments have shifted paradigms in a range of engineering disciplines, driving the transition from bulky, rigid constructions to flexible, lightweight integrated assemblies in areas such as biorobotics,^[^ ^6^ ^]^ haptics,^[^ ^7^, ^8^ ^]^ surgical technology,^[^ ^9^ ^]^ and microrobotics.^[^ ^10^ ^]^ Magnetic fields have been extensively studied as one of the principal ways of actuating such soft machines.

Magnetic soft machines (MSMs) consist of soft elements with programmable magnetic properties, such as magnetoresponsive polymers^[^ ^11^ ^]^ and small‐scale electromagnetic coils.^[^ ^12^ ^]^ The response of these elements to magnetic fields can mediate multitudinous mechanical behavior of MSMs, ^[^ ^13^, ^14^ ^]^ while allowing them to retain simple structure,^[^ ^15^, ^16^, ^17^, ^18^ ^]^ permitting laser machining ^[^ ^19^, ^20^ ^]^ and assembly.^[^ ^21^, ^22^ ^]^

The magnetization profile of an MSM determines the response in a magnetic field. In particular, the actuation response of magnetoresponsive polymers can be programmed by magnetizing in molds,^[^ ^23^, ^24^ ^]^ printing magnetic domains,^[^ ^25^, ^26^ ^]^ magnetic reprogramming,^[^ ^27^, ^28^, ^29^ ^]^ or programming of magnetic voxels.^[^ ^30^ ^]^ Generally, MSMs are designed to operate in magnetic fields while taking advantage of surrounding field topology.

Far‐field MSMs operate in magnetic fields generated by arrays of electromagnets or permanent magnets at distances up to a few tens of centimeters.^[^ ^31^, ^32^, ^33^ ^]^ These MSMs can operate as untethered^[^ ^34^, ^35^, ^36^ ^]^ or tethered devices,^[^ ^37^, ^38^, ^39^ ^]^ made of magnetoresponsive polymers or small electromagnetic coils.^[^ ^40^ ^]^ Although far‐field MSMs are highly mobile within their workspace, the workspace portability is tied to that of the magnetic field generation systems, which are generally bulky and heavy systems with limited portability. Consequently, far‐field MSMs are often proposed for applications where workspace portability is less of a concern and miniaturization and untethered operation is advantageous, such as for biomedical applications^[^ ^41^, ^42^ ^]^ as grippers,^[^ ^30^, ^43^ ^]^ muscles,^[^ ^44^ ^]^ stents,^[^ ^45^ ^]^ guidewires,^[^ ^46^ ^]^ and drug‐delivery devices.^[^ ^22^, ^47^ ^]^

Although proven to be challenging, there are active explorations aiming to realize independent and collaborative control of multiple far‐field MSMs, including modulating phase or frequency response,^[^ ^13^, ^30^, ^48^, ^49^, ^50^ ^]^ shape‐based magnetic anisotropy,^[^ ^51^, ^52^ ^]^ stiffness variability,^[^ ^53^ ^]^ or manipulating the field topology^[^ ^54^, ^55^ ^]^ at the cost of system complexity.^[^ ^56^ ^]^ In the latter case, independent control of far‐field MSMs, which are generally actuated with magnetic torques, requires 3 degrees of freedom (DOF) per effector when gradients are considered negligible.^[^ ^31^ ^]^ Consequently, although large coils can have relatively low resistance,^[^ ^57^ ^]^ the increasing number of required coils and needed driving currents (scaling with distance) for independent control of far‐field MSMs^[^ ^54^ ^]^ result in high power consumption.

Alternatively, near‐field MSMs bring field source and effector in direct vicinity. For example, permanent magnets are used for local magnetization and attraction of magnetorheological^[^ ^58^, ^59^ ^]^ and ferrofluidic robots,^[^ ^60^ ^]^ or combined with liquid metal‐based coil effectors to produce soft fish‐tail actuators.^[^ ^61^, ^62^ ^]^ However, these permanent magnet sources are stiff, require mobility to modulate the field, or require electromagnetic effectors which are challenging to miniaturize. Alternatively, electromagnetic near‐field sources generate time‐varying fields. For example, copper solenoid arrays control planar motion of ferrofluidic robots,^[^ ^63^ ^]^ liquid metal‐based solenoids exert linear forces on a permanent magnetic core for biomimicry^[^ ^64^ ^]^ and push and pull permanent magnets as part of a soft gripper.^[^ ^65^ ^]^ Also, printed circuit board‐based coil arrays control diamagnetically levitating permanent magnets,^[^ ^66^, ^67^ ^]^, direct sliding motion of disk‐shaped permanent magnets^[^ ^68^, ^69^, ^70^, ^71^ ^]^, and control the position of superparamagnetic nanoparticles.^[^ ^72^ ^]^ Finally, planar coils deflect uniformly‐magnetized membranes for microfluidic applications.^[^ ^73^ ^]^

In this study, we propose magnetoresponsive polymer effectors tailored to the nonhomogeneous axial and radial distribution of electromagnetic near‐fields generated by ultrathin flexible coils. There are several advantages to this approach. First, the combination of electromagnetic near‐field sources with magnetoresponsive polymer effectors combines the miniaturization ability and magnetic programmability of magnetoresponsive polymers with the temporal control of electromagnetic near‐fields. Second, the flexibility of both effector and coil allows integration of the MSM onto flexible portable surfaces. Third, the localized near‐field permits independent actuation of MSMs in an array in close proximity. Additionally, model‐based optimization of magnetoresponsive polymer effectors improves actuation response and allows utilization of the full functional surface area of the coils. A comparison between different electromagnetic near‐field MSMs is presented in **Table** **1** to place this work in the context of the state of the art.

**Table 1:** Comparison of near‐field actuators comprising stationary electromagnetic sources with mobile effectors. For multi‐layer printed circuit board‐based sources,^[^ ^66^, ^67^, ^68^, ^69^, ^70^, ^71^ ^]^ we have taken the distance between traces to be 0.2 mm for approximating thickness, unless reported otherwise. Effectors are reported in terms of type, thickness, magnetization profile, and motion. For the combined sources and effector the number of utilized sources per effector are shown. Also, work densities are listed, normalized with respect to currents. Values marked (‐) are not reported in the respective works. Listed values are approximations where the indication “≈” has been omitted for compactness

References	Electromagnetic near‐field source				Effector				Actuator	
	Thickness [µm]	Field [mT]	Gradient [mTmm^−1^]	Flexible	Type	Thickness [µm]	Magnetic profile	Motion	Sources [#]	Work Density [kJm^−3^A^−1^]
[63]	30· 10^3^	6.7	–	no	fluid	–	adaptive	planar	25	–
[64]	10· 10^3^	–	–	yes	permanent	6· 10^3^	uniform	linear	1	0.056
[65]	4 · 10^3^	1.5	–	yes	permanent	700	uniform	linear	2	2.5· 10^−4^
[66]	803	–	–	yes	permanent	500	uniform	planar	–	–
[67]	540	–	–	no	permanent	400	uniform	planar	48	–
[68]	470	0.1	–	no	permanent	500	uniform	planar	–	–
[69]	450	0.5	2	no	permanent	500	uniform	planar	128	–
[70]	378	0.5	2	no	permanent	790	uniform	planar	64	–
[71]	850	1	0.7	no	permanent	500	uniform	planar	64	–
[73]	376	0.8	–	no	polymer	170	uniform	linear	1	8 · 10^−5^
This	50	2	1	yes	polymer	30	nonuniform	programmable	1	0.3

We report such near‐field MSMs that combine single‐layer planar coils with magnetoresponsive polymer effectors (henceforth called “effectors”). These flexible, ultrathin (80–300 µm), lightweight (100 gm^−2^, including effector and coil), low power demanding (down to 500 mW), and fast (25 Hz) machines that operate with near‐fields and gradients on the order of 2 mT and 1 Tm^−1^, respectively. We demonstrate near‐field model‐based computation of task‐specific magnetization profiles on the basis of simulated exerted torques and forces. This methodology in combination with jig‐based magnetization and assembly‐based fabrication produces effectors capable of lifting, tilting, pushing, pulling, and grasping. Further, we show operation of near‐field MSMs in both air and aqueous environments, independent actuation in close proximity, synergistic operation, and integration on portable flexible curved surfaces to provide functionalization.

## 2 Results

**Figure 1:**
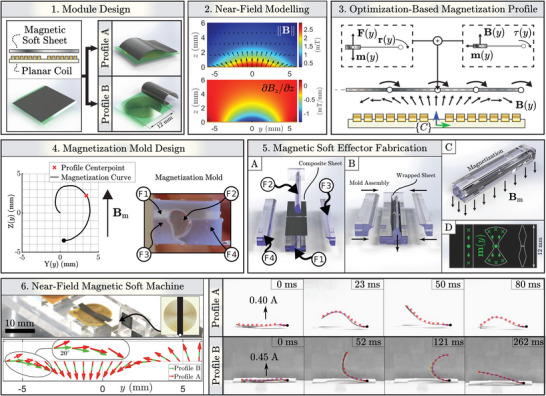
Design and fabrication of near‐field magnetic soft machines (MSMs). 1) Near‐field MSMs consist of magnetic soft elements as effectors located in proximity to a flexible planar coil substrate. Effectors can have different magnetization profiles that determine their deformation in response to the magnetic near‐field of the coil. 2) Magnetic near‐field profile $\left(B\in R^{3}\right)$ and axial gradients (∂*B*_*z*_/∂*z*) in the *yz*‐plane above the coil surface produced by a current of 1 A. 3) Effectors lay flat on the coil in their reference configuration. The magnetization profile $\left(m\left(y\right)\in R^{3}\right)$ along the coil diameter determines the experienced force $\left(F\left(y\right)\in R^{3}\right)$ and torque $\left(\tau \left(y\right)+r\left(y\right)\times F\left(y\right)\in R^{3}\right)$ due to the near‐field (B(y)), where r(y) represents a moment arm. 4) For each desired magnetization profile of an effector an associated unique 2D magnetization curve (Y(*y*), Z(*y*)) is computed based on a known magnetization field $\left(B_{m}\right)$ (for more detail see Figure S5, Supporting Information, and Experimental Section). Extruding the magnetization curve gives a 3D magnetization mold (F1–4). 5) Non–magnetized sheets are wrapped inside the mold and magnetized (A–C) within the magnetizing field (***B***_m_) to gain their desired magnetization profile. Post‐magnetization the sheets have a non‐uniform and uniform magnetization (D) along their short and long axis, respectively. The sheets are laser‐cut to form (parts of) effectors. 6) Coil and effector are assembled as a near‐field MSM. Effector magnetization profile determines deflection response (also see Figure S7, Supporting Information). The deflection responses of two effectors with different magnetization profiles (A,B) are shown. Both profiles are optimized for clockwise bending torque, with profile B constraining τ(*y*) to be in the direction of bending.

The proposed near‐field MSMs comprise two functional elements: A flexible planar spiral electromagnetic coil and a polymeric magnetic soft effector (**Figure** **1**.1). The magnetic soft effectors are considered parallel to the coil in its direct vicinity (<1 mm). The function of each effector is mediated by deformation in response to the magnetic near‐field $\left(B\in R^{3}\right)$ generated by the current running through the coil (Figure 1.2). We represent the near‐field using a multipole expansion model fitted to field measurements within a volumetric sweep obtained above the coil surface. The magnetic field is provided with respect to the coil center reference frame ({C}, Figure 1.3) and depends on the geometry of a particular coil design. In this work, we use spiral copper coils with an outer radius of R_o_ = 6 mm, inner radius of R_i_ = 1 mm, Cu wire thickness of 9 µm, and resistance of 12 Ω, attached to a thin Polyimide foil of thickness 25 µm (Figure S1, Supporting Information).

### 2.1 Magnetic Near‐Field

The nonhomogeneous magnetic near‐field $\left(B\in R^{3}\right)$ generated by the coils at a unit current (*I* = 1 A) reaches magnitudes up to 2 mT, scaling linearly with operating current (Figure 1.2). Gradients in the near‐field are on the order of 1 Tm^−1^ (Figure S2, Supporting Information). At steady operating currents of *I* = 0.2 A the magnetic near‐field and gradient reaches 0.4 mT and 0.2 Tm^−1^, respectively, with a power consumption of 0.5 W. This power consumption is at least two orders of magnitude smaller than the wattage necessary for producing similar gradients with magnetic far‐fields generated by large iron‐cored electromagnets at conventional operating distances.^[^ ^57^ ^]^

### 2.2 Magnetic Soft Effectors

Motion of magnetic effectors within the magnetic near‐field generated by the coil results from exerted distributed forces ($F\in R^{3}$) and torques ($\tau \in R^{3}$), determined by the magnetization profile ($m\in R^{3}$) of the effector. For application‐specific optimization of the magnetization profile we consider the effector as a parametric surface r(α,z)=(rcosα,rsinα,z) parallel to the coil, where α ∈ [0, 2π], *r* ∈ [0, *R*] spans the radius of the coil, and $z\in R^{+}$ is the distance between the coil and effector.

Effectors are obtained by assembly of strips of pre‐magnetized magnetic composite elements, which are laser‐cut from magnetized sheets. The sheets are based on polydimethylsiloxane (PDMS) and hard‐magnetic microparticles with a volumetric ratio of 0.35 (Figure S3, Supporting Information). Here, we use a sheet thickness within the range of 30–400 µm depending on desired MSM functionality. This assembly method, as well as axial symmetry of the magnetic near‐field, simplifies computation of the magnetization profiles to one dimension, reducing the parametric surface to a straight curve (r(α,z)→(0,y,z)) with *y* ∈ [0, 2*R*] spanning the coil diameter. The magnetization profile of each element (m(y)) is computed assuming their unactuated planar configuration (refer to Section S1 and Figure S4, Supporting Information). The operation of each element is dictated by the acting distributed forces (F(y)=∇(B(y)·m(y)) and torques (τ(y)=m(y)×B(y)) in the magnetic near‐field of the coil (Figure 1.3).

A homogeneous magnetic field (∥B∥= 2T) is used for magnetization of the magnetic composite sheets (Figure 1.4). Combining the desired magnetization profile (m(y)) of the sheets with the external field, a relative magnetizing angle $\left(\theta \left(y\right)=∠\left(m\left(y\right),B_{m}\right)\right)$ is computed. The relative magnetizing angles of the sheet across the coil diameter are used to compute a 2D magnetization curve, providing the desired magnetization profile of sheets when subjected to $B_{m}$ (Figure S5, Supporting Information). From the 2D curve, a 3D mold is fabricated for sheet magnetization (Figure 1.4 F1–4). The general structure of the mold (Figure 1.5 A–C) facilitates folding of the composite sheets, permitting precise high‐throughput and time‐efficient magnetization, and providing a non‐uniform and uniform magnetization along the short and long axis of the sheet, respectively (Figure 1.5 D). The non‐uniform magnetization profile along the short axis of the sheets are validated with stray field measurement‐based reconstruction (see Section S2 and Figure S6, Supporting Information).

The final magnetic effectors are assembled using kirigami‐based techniques. Laser engraving is used to cut desired shapes from the magnetized sheets, constituting magnetic composite elements as building blocks for the effectors (Figure 1.5 D). We demonstrate our framework using two elementary MSMs. The magnetic effectors constitute a continuous strip fixed to the coil substrate on one side (Figure 1.6). Interaction between the near‐field and magnetized material within the effectors produce bending moments cause C‐ and S‐shaped deflection directly upon coil activation (Movie S1, Supporting Information). Using a high‐speed camera in conjunction with an autonomous tracker the effectors are capable of moving with a maximum speed of 0.4 ms^−1^ and reach a steady state at 100–150 ms (Figure S7, Supporting Information). This dynamic response of the effector can be modified by activating the MSM with a different temporal profile of the magnetic near‐field.

### 2.3 Individual Magnetic Soft Machines

**Figure 2:**
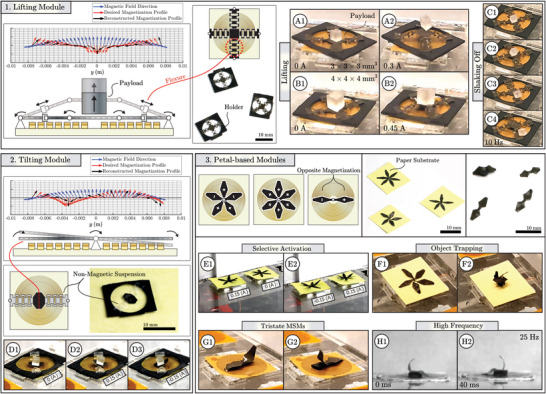
Examples of individual near‐field magnetic soft machines (MSMs). 1) Lifting MSMs have magnetization profiles optimized for lifting forces at the center and bending moments on the connecting arms. Flexures are laser‐cut on the arms to permit stretching. Payloads of 27 mg (A1,2) and 64 mg (B1,2) are lifted by the MSM weighing 15 mg, comprising coil and effector. High‐bandwidth vibratory motion (>10 Hz) facilitates shaking off payloads (C1–4). 2) Tilting MSMs maximize torque about their transverse axis. The effectors are suspended above the coil by a non‐magnetic polydimethylsiloxane (PDMS) strip containing flexures to enable stretch. These MSMs are able to tilt, for example, a payload, here a 27 mg cube (D1–3). 3) Petal‐shaped elements are fixated on the coil center and have magnetization profiles that optimize bending moments at positive or negative coil current. Effectors can be activated selectively while being in close proximity, depending on current direction (E1,2). Assemblies of identical petals show axisymmetric gripper‐like deformation for object trapping (F1,2). Oppositely‐magnetized petals can be combined to make tristate MSMs, which have different configurations at positive, negative, and no current (G1,2). Rapid phase shift in coil current allows effector motion at high frequency (H1,2).

The current running through the electromagnetic coil of an MSM provides control over one DOF of the magnetic effector. Effectors can be cut and assembled with varying structural complexity using kirigami‐based techniques. These effectors can be designed to lift, tilt, grab, and pull/push by modifying the effector magnetization profile. We demonstrate this in a study employing effectors cut out of magnetic polymer sheets of thickness 30 µm with application‐specific magnetic moment profiles (**Figure** **2**).

Lifting effectors employ a symmetric magnetization profile optimized for maximum vertical force at the coil center, as well as bending moments at the boundary. These MSMs are capable of vertical lifting of a payload (Figure 2.1 and Movie S2, Supporting Information). The lifting MSMs are assembled from two strips of magnetic composite soft elements placed perpendicular to one another and connected in the center. Flexures are laser cut in the effectors to enable stretching during vertical motion. The resulting MSM, constituting both coil and effector, has a mass of 15 mg and is able to lift payloads of 27 mg (Figure 2.1 A1,2) and 60 mg (Figure 2.1 B1,2) at coil currents of 0.2 A and 0.45 A, respectively. The payloads are 3D printed cubes with dimensions 3 × 3 × 3 mm^3^ and 4 × 4 × 4 mm^3^. Additionally, high‐bandwidth motion of the lifting MSMs (>10 Hz) permits shaking off payloads (Figure 2.1 C1–4). The stroke length of the lifting effector is approximated at 2 mm for the 27 mg (26.5 · 10^−5^ N) cube in Movie S2 (Supporting Information). Considering the approximate volume of the coil (⌀ 12 × 0.06 mm^3^) and effector (⌀ 12 × 0.03 mm^3^), the corresponding work density is on the order of 0.052 kJm^−3^. Similarly, considering the MSM mass of 15 mg the corresponding work capacity of the lifting MSM is on the order of 0.35 · 10^−4^ kJ kg^−1^. These values are for coil currents of 0.2 A. For currents of 1 A the work density and capacity scale linearly to 0.29 kJ m^−3^ A^−1^ and 1.2 · 10^−4^ kJ kg^−1^ A^−1^, respectively.

Tilting effectors have a magnetization profile optimized for torque about their central axis (Figure 2.2). These effectors are suspended over the coil center using a non‐magnetic PDMS flexure‐based segment. Such MSMs constitute a lightweight miniaturized tilt table to manipulate the orientation of cargo (Figure 2.2 D1,2 and Movie S3, Supporting Information). Tilting effectors respond oppositely to positive and negative coil currents.

Gripper‐like effectors are assembled from subcomponents referred to as petals, which can employ different magnetization profiles and be activated selectively (Figure 2.3 E1,2). These petals have a magnetization profile that maximizes the bending moment with respect to their point of attachment on the coil center (Movie S4, Supporting Information). Combining petals with similar magnetization profiles results in self‐locking effectors suitable for object trapping (Figure 2.3 F1,2). The gripper does not reopen due to the combination of attractive forces in the locked state and the petals deflecting beyond the near‐field. Finally, petals with dissimilar or opposite magnetization profiles may be combined to produce effectors with functional responses at different coil polarities, providing tristate MSMs (Figure 2.3 G1,2). Additionally, the petal‐based MSMs are capable of periodic motion at frequencies reaching 25 Hz (Figure 2.3 H1,2).

### 2.4 Arrays of Magnetic Soft Machines

**Figure 3:**
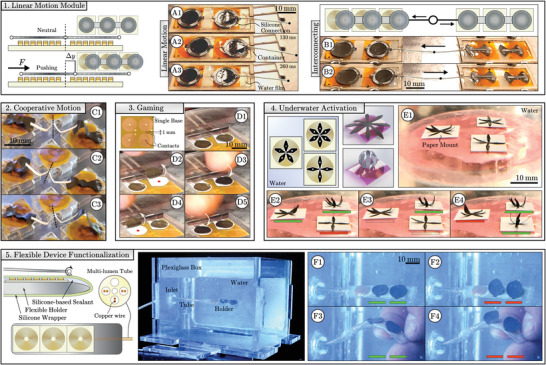
Magnetic soft machines (MSMs) as magnetic soft actuator array systems. 1) Interconnected effectors as soft muscles with planar linear force‐optimized magnetization profiles. 2) Deflecting MSMs are interconnected to tilt a connecting element: a pre‐bent paper strip. 3) Proximal positioning and independent actuation of effectors at non‐equal time instants can be used for human interaction as a game. 4) Different effectors can be placed in proximity to each other and activated underwater, potentially permitting underwater applications. The bars underneath effectors indicate coils running positive (green), negative (red), or no (empty) current. 5) MSMs may be arranged on flexible or curved holders. Coils and effectors are separated by a fluid‐impermeable membrane, allowing effectors to operate while shielding the coil from direct contact with a surrounding fluid.

Individual MSMs may be arranged as arrays with the possibility to be interconnected (**Figure** **3**). Due to the drop‐off in the near‐field magnitude of the coil beyond its radial edge, the proximally positioned MSMs can be independently controlled.

Radial linear motion MSMs contain effectors that displace in the radial direction upon coil activation (Figure 3.1 and Movie S5, Supporting Information). The effectors carry magnetization profiles that are optimized to experience magnetic forces in the radial direction on the coil surface, with the achievable forces being quantified in simulation (Figure S8, Supporting Information). Notably, radial forces scale nonlinearly with effector thickness and displacement from the coil center, and linearly with magnetic volumetric ratio and coil current. The maximum stroke length of these effectors is approximately equal to the coil radius (*R*), that is a forward and backward stroke of *R*/2 constituting push/pull action. For a specific case of effectors with a thickness of 0.6 mm, magnetic volumetric ratio of 0.35, and coil current of *I* = 1 A, the maximum work density and work capacity are on the order of 0.3 kJ m^−3^ A^−1^ and 1·10^−4^kJ kg^−1^ A^−1^, respectively. Interconnection of these effectors for collaborative pulling and pushing action additively improves the achievable forces upon simultaneous activation. Alternatively, the linear motion MSMs can be positioned in an array and actuated independently for stand‐alone or collaborative action (Figure S9 and Movie S5, Supporting Information).

Further, interconnected MSMs may also be selectively activated to achieve motion of an interconnecting structure, unable to be performed by individual MSMs (Figure 3.2). Here, simultaneous antagonistic or agonistic activation of MSMs results in tilted or level orientation of the connecting element, respectively (Movie S6, Supporting Information). Interconnecting multiple similar or dissimilar MSMs can thus provide multi‐DOF motion to connecting bodies.

Alternatively, arrays of individual MSMs and their independent activation can be used for human interaction (Figure 3.3). Selective activation at non‐equal time instants as well as their durability and safety, can be used for the realization, for example, of interfaces intended for gaming, rehabilitation, and concentration practice (Movie S6, Supporting Information).

**Figure 4:**
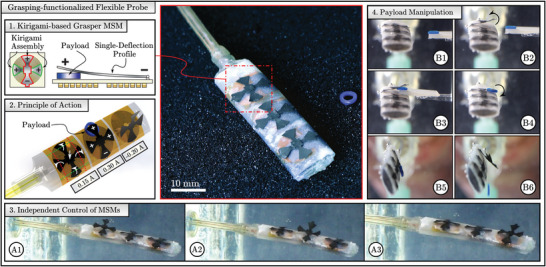
A flexible tip functionalized for payload grasping and release using near‐field magnetic soft machines (MSMs). 1) The tip utilizes three independent grasping effectors, each assembled from single‐deflection magnetic soft elements using kirigami‐based technique. 2) Opening and closing of each effector is independently controlled by current within the respective planar coils. 3) Independent control of MSMs is demonstrated by actuating each effector using phase‐shifted current inputs of 0.2 A. 4) The tip‐mounted MSMs are used to grab and release a payload (20 mg).

Finally, proximal MSMs can be activated within aqueous environments (Figure 3.4). By contrast, selective activation of conventional far‐field MSMs arranged in close proximity would require magnetic shielding or multi‐DOF electromagnetic systems. The flexibility of the coils permits integration and functionalization of flexible instruments (Figure 3.5 and Movie S7, Supporting Information). Coils are applied on a flexible 3D‐printed holder. A thin (200 µm) rubber sheet around the holder shields coils from direct contact with water. Coils transition from a planar to curved surface without loss of performance. Functionalization of a flexible probe tip is demonstrated with an array of grasping MSMs (**Figure** **4**). The effectors are able to press a payload between themselves and the coil surface (Figure 4.1). Payload transport and release is mediated by the direction of current through the coils (Figure 4.2). The flexible tip is designed to operate within a fluidic environment, shielding the coil from direct contact while allowing independent control of effectors (Figure 4.3). The effectors are able to hold a payload (mass 20 mg) against the direction of gravity and release on demand (Figure 4.4). This demonstrates that the presented technology enables development of portable flexible devices with arrays of independently controlled actuators.

## 3 Discussion

This study reports operation of optimally‐magnetized magnetoresponsive polymer effectors in the nonhomogeneous magnetic near‐field generated by planar single‐layer coils. By combining the microscale thickness manufacturing of coils and effectors, as well as utilizing a near‐field localized to the surface area of the coils, selectively actuable and densely packed devices can be realized at scales that are currently infeasible for otherwise actuated structures, such as tendons or hydraulics. Based on the axial and radial distribution of the near‐field and gradients, effector magnetization profiles are tailored to maximize torques or forces. We demonstrate the near‐field MSMs as axial and radial linear actuators, tilting devices, grippers, gaming tools, and functional elements on flexible curved surfaces in air and aqueous environments.

The presented magnetic soft effectors expand the achievable motion of near‐field MSMs seen in literature thus far. For example, looking at the existing tethered systems in this field, the arms of grippers are collectively actuated on a thin single coil surface rather than requiring multiple adjacent coils.^[^ ^65^ ^]^ Similarly, densely packed arrays of individually actuable grippers can be achieved, with tradeoff in mobility.^[^ ^43^ ^]^ Tilting motion of planar polymer effectors is achieved instead of requiring electromagnetic fish‐tail effectors.^[^ ^61^ ^]^ Radial force‐optimized effectors provide directional control by a single coil for sliding, rather than requiring multiple coils to direct sliding permanent magnets with uniform magnetization.^[^ ^71^ ^]^ By utilizing the central and radial regions of the electromagnetic near‐field, the number of sources to actuate an effector is reduced to one. Consequently, arrays of near‐field MSMs with equal number of sources and effectors can be realized, scalable to the surface area of individual electromagnetic coils. Although coils with a diameter of 12 mm are used in this study, they can be scaled down to submillimeter scale.^[^ ^66^, ^67^, ^68^, ^69^ ^]^ Also, jig‐based magnetization of effectors has been shown at sizes as small as 500 µm.^[^ ^21^ ^]^

The fabrication of both coil and effectors at microscale thickness can be used to produce ultrathin muscle actuators, demonstrated as lifting and radial linear motion MSMs. Although the near‐field MSMs have relatively low work capacity and work density compared to other types of artificial muscles,^[^ ^44^ ^]^ they are capable of high‐bandwidth motion, push and pull action, large stroke compared to their thickness, as well as grid‐like arrangement and independent actuation. Although our work reports MSM operation at frequencies up to 25 Hz, higher bandwidths can be achieved with different electronic equipment. A comparison between the presented MSMs (lifting and radial linear motion) and other types of artificial muscles and near‐field magnetic actuators is shown in Table S1 (Supporting Information).^[^ ^44^, ^64^, ^65^, ^74^, ^75^, ^76^, ^77^, ^78^ ^]^

Muscle‐like actuators are the lifting and radial linear motion MSM. For the lifting MSM, effector displacement is in the axial direction of the coil. Therefore, field magnitude changes but direction remains relatively constant, resulting in decreasing but invariably directed forces on the effector. Comparatively, for radial motion MSM, near‐field direction changes but magnitude remains relatively constant, resulting in a shift from radial to axial forces with displacement relative to the coil center (Figure S8, Supporting Information). In both cases, forces (and torques) on effectors can be increased with coil current, or by increasing the volumetric ratio of magnetic microparticles and effector thickness, at the cost of bending stiffness and weight.^[^ ^37^ ^]^

Near‐field MSMs with effectors that deflect or displace in the axial direction of the coil benefit from impulse currents through the coils, whereas effectors operating in‐plane can be operated with both impulse and step currents. This follows from the properties of the near‐field, as field and gradient magnitudes approach zero at the radial edge of the coil and beyond a few millimeters above the surface. Increasing coil current scales power consumption quadratically, and power consumption of a near‐field MSM array scales linearly with number of coils. The power consumption of single coils with resistance of 12 Ω and currents of 0.2–1 A used in this work ranges between 0.5 and 12 W. Within air at room temperature and with constant operating currents at and below 0.2 A (0.5 W) we did not experience overheating of the coil, which is equivalent to providing impulse currents of 1 A for 40 ms per second, without active cooling. For aqueous operating environments (Figure 4), heat dissipation is improved compared to air, which allows to operate coils continuously at 0.3 A.

An increase in permissible operating current of the planar coils, and consequently the achievable magnetic field and gradient magnitudes, can be achieved by increasing the cross‐sectional surface area of the copper wires. By reducing the current density and power consumption of the coil by increasing width and simultaneously decreasing spacing of the wires, as well as increasing thickness. Notably, both wire thickness and width linearly decrease coil resistance and thereby power consumption, whereas elevated current increases coil power quadratically but magnetic field magnitude linearly.

The magnetic field generated by the planar coils approaches azimuthal symmetry due to their spiral shape. Therefore, the magnetic field can be modeled analytically with a multipole expansion model (see Experimental Section). In our work, the geometrical properties of the coil are not subject to optimization and chosen according to the available wet etching‐based coil fabrication process (see Experimental Section). The resulting magnetic field topology is used as the basis to design the magnetic soft effectors. In further work, by including coil geometry in the MSM design process, task‐specific variations in magnetic field topology can be achieved. For example, individually controllable perpendicular conductors permit generating propagating magnetic field profiles.^[^ ^72^ ^]^ Therefore, by extension of the presented work, both coil and effector can be designed simultaneously and co–dependently.

We envision that near‐field MSMs can find application in healthcare, electronics, fluidics, and optics. For example, radial linear actuators for needle biopsy,^[^ ^79^ ^]^ deflecting MSMs for concentration practice and hand movement during stroke rehabilitation,^[^ ^80^ ^]^ axial (lifting) linear actuators as electrical switches or programmable valves,^[^ ^73^ ^]^ and tilting surfaces for orienting mirrors or lenses.^[^ ^30^ ^]^ Additionally, J‐heating of coils can be utilized to fabricate magneto‐thermal responsive effectors.^[^ ^81^ ^]^ Finally, hard‐magnetic particles in effectors as used in this work can be replaced by soft‐magnetic particles that enable remagnetization of effectors, and utilizing magnetic anisotropy of effector shape to control directions of magnetization easy axes across the effector body.^[^ ^11^, ^17^, ^82^, ^83^ ^]^ Although the field of planar coils could be insufficiently strong to remagnetize soft‐magnetic particles for macroscale material responses of composite‐based effectors, far‐fields can be used to appropriately tailor their magnetization profile.^[^ ^82^ ^]^ Subsequently, these remagnetized soft‐magnetic effectors can interact with the near‐field gradients of planar coils for exertion of forces. In this case, the relatively high magnitude of fields and gradients in near‐ and far‐fields, respectively, may be utilized. As an extension, due to the different near‐ and far‐field properties the collaborative operation of near‐ and far‐field MSMs can be investigated further. This gives even more freedom to the design of MSMs and enables further application in, for example, minimally invasive surgical devices, independently‐activated components in soft electronics, and small‐scale local actuators in soft robotics.

## 4 Experimental Section

### Fabrication of Flexible Planar Coils

The planar electromagnetic coils were prepared using polyimide copper laminate (DuPont Kapton, IM30‐LM‐000110, Goodfellow GmbH, Germany) of 25 µm thickness with 9 µm thick copper coating (Figure S1, Supporting Information). Image‐reversal optical lithography and wet etching was used to pattern the coil on the laminate. First, the copper surface was cleaned using O_2_ oxygen plasma for 2 min. AZ5214e image reversal photoresist (MicroChemicals GmbH, Germany) was spin coated at 1000 rpm for 30 s and baked at 110 °C for 2 min. The planar coil design was patterned using a UV laser writer (DWL66, Heidelberg Instruments, Germany) with a wavelength of 410 nm, post baked at 120 °C, flood exposed under UV light, and developed in AZ351b developer. Subsequently, the laminate was post baked at 120 °C for 5 min and wet etched in 1:10 sodium persulfate solution in DI water (B327, AG TermoPasty, Grzegorz Gasowski, Poland) kept at 60 °C. Residual etching agent was removed with DI water. Photoresist was then removed with acetone, isopropanol, and DI water.

### Modeling of Magnetic Near‐Field

The magnetic near‐field generated by the coils at a constant operating current (*I* = 0.15 A) was measured with a Hall‐effect sensor (MLX 90371, Melexis, Ypres, Belgium) attached to an Arduino Due microcontroller board (Figure S2, Supporting Information), assigned local frame ({*E*}). The sensor was moved in a volumetric sweep above the coil surface with a six‐DOF Franka Emika robotic arm, assigned base frame {*B*}. Measurements were taken at different sensor positions $\left({}^{B}p\in R^{3}\right)$ with constant orientation ${(}_{E}^{B}R\in \mathrm{SO}\left(3\right))$. Local measurements taken by the sensor $\left({}^{E}B\left(I,{}^{B}p\right)\right)$ were compensated for the earth magnetic field $\left({}^{E}B_{0}\right)$ and transformed to the base frame,

(1) $${}^{B}B\left(I,{}^{G}p\right)={}_{E}^{B}R\left({}^{E}B\left(I,{}^{B}p\right)-{}^{E}B_{0}\right)$$

The coil center position $\left({}^{B}p_{C}\right)$ was initially approximated from visual inspection of the measurements. Thereafter the field measurements were represented with respect to the coil center $\left(\left\{B\left(I,p\right)\;|\;p={}^{B}p-{{}^{B}p}_{C}\right\}\right)$.

An *N* ^th^‐order Cartesian multipole‐expansion $\left(\Psi \left(p\right):R^{3}\to R\right)$ of the form

(2) $$\Psi \left(p\right)=\sum_{m=1}^{M}\sum_{n=1}^{N}b_{n}\cdot \frac{\partial^{n}}{\partial z^{n}}\frac{1}{\sqrt{x^{2}+y^{2}+{\left(z+d_{m}\right)}^{2}}}$$

was implemented symbolically in Matlab R2022. The derived near‐field model $\left(\tilde{B}:=\nabla \Psi \left(p\right)\in R^{3}\right)$ was fit to the measurements, where $\nabla :=\langle \frac{\partial }{\partial x},\frac{\partial }{\partial y},\frac{\partial }{\partial z}\rangle$ and 〈 · 〉 represents a column vector. Field source displacement variables $\left(d_{m}\in R^{+}\right)$ were added along the coil axis to resolve unboundedness of Ψ in the near‐field when *z* → 0. Scalar coefficients $\left(b_{n}\in R\right)$, model order $\left(N\in Z^{+}\right)$, and number and values of displacement variables $\left\{M\in Z^{+},d_{m}\right\}$ were determined using a parametric sweep and convex optimization with Matlab's fmincon. The near‐gradient field was obtained by symbolic differentiation of the near‐field model $\left({\tilde{B}}_{\nabla }:=\nabla^{T}\tilde{B}\in R^{3\times 3}\right)$. The field model was fit to measurements using linear least squares.

To improve the quality of fitting a near‐field model $\left(\tilde{B}\left(p\right):R^{3}\to R^{3}\right)$, the field measurements (B(I,p)) were adjusted for uncertainties in the coil center position and Hall‐effect sensor frame orientation. We consider an optimization vector $\left(o=\left\langle \delta x,\delta y,\alpha ,\beta ,\gamma ,d\right\rangle ,\;d\in R^{M}\right)$ such that p:=p−⟨δx,δy,0⟩ and $B\left(I,p\right):=R_{x}\left(\alpha \right)R_{y}\left(\beta \right)R_{z}\left(\gamma \right)B\left(I,p\right)$. The combination {*N*, *M*} minimizes

(3) $$\begin{array}{cc} O\left(N,M\right)=arg\;min_{o}\; & ∥\frac{\left(\tilde{B}\left(p\right)-B\left(I,p\right)\right)}{B{\left(I,p\right)}^{T}}∥\\ s.t.\; & d\geq 0,\;\forall d_{m} \end{array}$$

The objective function (*O*(*N*, *M*)) was chosen to equalize the influence of measurement‐model errors in regions where the residual error was low but relative error may be high, that is, the edges of the coil.

### Materials and Fabrication of Magnetic Soft Effectors

Magnetic effectors were produced by from post‐processing of vulcanized magnetic polymer composite (MPC) sheets. The MPC constitutes a suspension of polydimethylsiloxane (PDMS) (Sylgard 184, #101697, Farnell, UK) and Pr‐Fe‐Co‐Nb‐B microparticles (MQP‐16‐7‐11277‐070, Magnequench GmbH, Germany) with a mean diameter of 5 µm. The MPC suspension was made by mixing PDMS at a 10:1 volume ratio of base‐curing agent, followed by manual introduction of microparticles at a volumetric ratio of 0.35 (Figure S3, Supporting Information). We note that the mechanical properties of these composites were documented in previous works. In particular, for the chosen composition the shear modulus and shear storage modulus were on the order of 1.2 MPa and 77 kPa, respectively.^[^ ^37^, ^84^ ^]^

Molds for MPC sheets were made by laser engraving (Speedy 300, Trotec Laser, Marchtrenk, Austria) rectangular cavities with depth between 30 and 300  µm. The cavities were treated with a release agent (Ease Release 200, Smooth‐On Inc., USA). MPC mixture was poured inside and uniformly distributed using a Stanley knife. Finally, the MPC‐filled molds were vulcanized at 100 °C for 1 h to form sheets. The sheets were manually removed from the molds.

Magnetization of the sheets was determined based on desired magnetic torque and force distribution in the magnetic near‐field of the coil (Figure S4, Supporting Information). Magnetization was performed using a uniform magnetic field $\left(B_{m}\right)$ generated by a vibrating sample magnetometer (GMW 3474‐140, GMW, Redwood City, California). Non‐uniform magnetization profiles were achieved by wrapping sheets in 3D‐printed molds (Figure S5, Supporting Information). The mold geometry was determined from a 2D magnetization curve. We compute this curve by considering a reference configuration of a planar MPC sheet perpendicular to the direction of $B_{m}$. In addition, the sheet has a desired magnetization profile m(y), where *y* ∈ [0, 2*R*] spans the coil diameter. The magnetizing field and magnetization direction have an angular offset $\left(\theta \left(y\right)=∠\left(B_{m},m\left(y\right)\right)\right)$. The magnetization curve was then computed with forward Euler integration,

(4) $$\left[\begin{array}{c} Y(y)\\ Z(y) \end{array}\right]=\int_{0}^{y}\left[\begin{array}{c} \cos\theta (\sigma )\\ -\sin\theta (\sigma ) \end{array}\right]\;d\sigma$$

Extruding the magnetization curve provides a mold around which sheets were wrapped for magnetization. At this point the sheets have non‐uniform and uniform magnetization along their short and long axis, respectively. Finally, the sheets were laser‐cut along the short axis to form 1D non‐uniformly magnetized elements which were subsequently assembled with glue (Loctite 401) to form 2D magnetic soft effectors.

### Near‐field MSM Demonstrations

Demonstrations of near‐field MSMs in water were performed within a laser‐cut plexiglass box (Figure 3). Coils were shielded from water by covering with a plastic sheet (thickness 500 µm, Figure 3.1). Also, silicone rubber (thickness 200 µm) was wrapped around the coils attached to a flexible 3D‐printed holder (elastic resin, Formlabs Form 2). Current was supplied to the coils by copper wires running along a multi‐lumen polyurethane tube, attached to the base of the flexible holder. Water was used to reduce friction between coil and effector during demonstrations of linear motion (Figure 3.2). Interconnecting linear‐motion MSMs was done by gluing (Loctite 401) a laser‐cut silicone cover (thickness 200 µm) onto the effectors. Motion was constrained in a linear direction by a 3D‐printed holder (elastic resin, Formlabs Form 2).

### Calculation of Work Density and Work Capacity

Work density and capacity were calculated for the lifting and radial linear motion MSMs. Each MSM consists of a polyimide foil, copper coil, and a magnetoresponsive polymer effector. For the calculation of work density and work capacity, all components were simplified as circular structures with outer radius (*R*_o_).

Further, the polyimide foil has thickness (*T*_f_ = 41 µm), density (ρ_f_ = 1420kgm^−3^), volume $\left(V_{f}=\pi R_{o}^{2}T_{f}\right)$, and mass (*M*_f_ = *V*_f_ρ_f_). The copper coil has thickness (*T*_c_ = 9 µm), density (ρ_c_ = 8960kgm^−3^), wire filling factor (α = 0.5), volume $\left(V_{c}=\pi R_{o}^{2}T_{c}\right)$, and mass (*M*_c_ = *V*_c_ρ_c_α). Finally, the effector has thickness (*T*_e_) which varies between MSMs, magnetic volumetric ratio (ϕ = 0.35), density of PDMS (ρ_e, 1_ = 965kgm^−3^), density of magnetic microparticles (ρ_e, 2_ = 7500kgm^−3^), volume $\left(V_{e}=\pi R_{o}^{2}T_{e}\right)$, and mass (*M*_e_ = *V*_e_((1 − ϕ)ρ_e, 1_ + ϕρ_e, 2_)). Therefore, each MSM has volume and mass

(5) $$\begin{array}{cc} V_{\mathrm{MSM}} & =V_{f}+V_{c}+V_{e} \end{array}$$

(6) $$\begin{array}{cc} M_{\mathrm{MSM}} & =M_{f}+M_{c}+M_{e} \end{array}$$

For lifting MSMs the work density and capacity were approximated from Movie S2 (Supporting Information) (also see Figure 2.1 A1,2). A lifting MSM with effector thickness (*T*_e_ = 30 µm) and running current (*I* = 0.2 A) lifts a 27 mg payload (weight *W*_p_ = 26.5 · 10^−5^ N) an approximate distance $\left(D=2\cdot 10^{-3}\;m\right)$ from the unactuated neutral position. Work density (*WD*_lift_) and capacity (*WC*_lift_) of lifting MSMs was then calculated as

(7) $$\begin{array}{cc} WD_{\mathrm{lift}} & =W_{p}D/\left(V_{\mathrm{MSM}}I\right) \end{array}$$

(8) $$\begin{array}{cc} WC_{\mathrm{lift}} & =W_{p}D/\left(M_{\mathrm{MSM}}I\right) \end{array}$$

For radial linear motion MSMs the work density and capacity were computed in simulation. An effector of thickness (*T*_e_ = 600 µm) was positioned a distance (Δ*z* = 200 µm) above the coil (see Figures S8 and S9, Supporting Information). The magnetization profile of the effector was optimized for radial (*y*‐direction) forces. The effector was divided in infinitesimal volumes (*dV* = *dxdydz*) with magnetic moment (m(x,y,z)=M(x,y,z)dV), where $M\in R^{3}$ represents the magnetization vector, $∥M∥=B_{r}V_{e}\phi /\mu_{0}$ was the magnetization magnitude, *B*_r_ = 1 T was the remanence of the here used Pr‐Fe‐Co‐Nb‐B microparticles, and µ_0_ was the magnetic vacuum permeability. Then, the magnetic force $\left(F=\left\langle F_{x},F_{y},F_{z}\right\rangle \right)$ on the effector at a radial displacement (Δ*y*) based on the magnetic field model $\left(\tilde{B}\right)$ of the coil was given by

(9) $$F\left(\Delta y\right)=\;\int \int \int \;\left(M(x,y,z)\cdot \nabla \right)\tilde{B}\left(x,y+\Delta y,z+\Delta z\right)\;dV$$

The radial force profile (*F*_*y*_(Δ*y*)) was symmetric with respect to the neutral position (Δ*y* = 0) (Figure S8C, Supporting Information). Therefore, assuming no friction, a load of magnitude *F*_load_ = *F*_*y*_(Δ*y*) can be displaced a maximum distance of 2Δ*y* (Figure S8E, Supporting Information). Then, the maximum work density (*WD*_radial_) and capacity (*WC*_radial_) of the radial linear motion MSM was calculated as

(10) $$\begin{array}{cc} WD_{\mathrm{radial}} & =arg\;max_{\Delta y}\left(2F_{y}\left(\Delta y\right)\Delta y\right)/\left(V_{\mathrm{MSM}}I\right) \end{array}$$

(11) $$\begin{array}{cc} WC_{\mathrm{radial}} & =arg\;max_{\Delta y}\left(2F_{y}\left(\Delta y\right)\Delta y\right)/\left(M_{\mathrm{MSM}}I\right) \end{array}$$

### Control and Power Systems

Coils were powered by three dedicated iPOS3602 VX‐CAT drives (Technosoft S.A., Neuchtel, Switzerland). The drives were connected to an external research laptop running Linux Ubuntu 18.04 through EtherCAT network. Selective current control was achieved via a custom‐made interface implemented in C++. Current profiles used during experimental demonstrations of MSMs represent a series of impulse, step, and square waveforms. The current used during near‐field MSM activation was limited to a maximum of 1 A.

## Conflict of Interest

The authors declare no conflict of interest.

## Author Contributions

M.R. and J.S. conceived the project and managed the research. M.R. fabricated the magnetic composite sheets. J.S. fabricated the magnetic effectors, and performed and recorded experiments. P.M. and Y.Z. designed and fabricated planar electromagnetic coils. M.R. provided the magnetic near‐field model. P.M. performed SEM and VSM characterization of the magnetic composites. P.M. carried out magnetic stray field imaging and analysis supported by Y.Z. and D.M. Additionally, P.M. and D.M. fabricated the planar electromagnetic coils. M.R. compiled figures and videos supported by J.S., V.K.V., P.M., D.M., and S.M. Also, M.R., J.S., P.M., V.K.V., D.M., and S.M. participated in discussions on the selection of demonstrators and development of the concept of using near‐fields for actuation of magnetic soft robots. Finally, M.R. and J.S. wrote the manuscript with contributions from all authors. D.M. and S.M. supervised this project.

## Supporting information

Supporting Information

Supplemental Movie 1

Supplemental Movie 2

Supplemental Movie 3

Supplemental Movie 4

Supplemental Movie 5

Supplemental Movie 6

Supplemental Movie 7

## Data Availability

The data that support the findings of this study are available from the corresponding author upon reasonable request.
