# Supplementary material for: Locally Addressable Energy Efficient Actuation of Magnetic Soft Actuator Array Systems
Source: Adv Sci (Weinh). 2023 Jun 17;10(24):2302077. doi: 10.1002/advs.202302077 (PMC10460866; doi:10.1002/advs.202302077)
Supplement: Supplementary file 1 — Supporting Information [file ADVS-10-2302077-s001.pdf]

ADVANCED SCIENCE
Open Access

Supporting Information

for *Adv. Sci.*, DOI 10.1002/advs.202302077

Locally Addressable Energy Efficient Actuation of Magnetic Soft Actuator Array Systems

Michiel Richter, Jakub Sikorski, Pavlo Makushko, Yevhen Zabila, Venkatasubramanian Kalpathy Venkiteswaran, Denys Makarov and Sarthak Misra**

Supplementary Materials:
Locally Addressable Energy Efficient Actuation of Magnetic Soft Actuator Array Systems

Michiel Richter, Jakub Sikorski, Pavlo Makushko, Yevhen Zabila,
Venkatasubramanian Kalpathy Venkiteswaran, Denys Makarov,
and Sarthak Misra

The PDF file includes:

Text

Fig. S1. Geometry of planar electromagnetic coils on flexible substrates.

Fig. S2. Measurement setup, measurements, and model for the magnetic near-field.

Fig. S3. Fabrication and characterization of magnetic composite soft sheets.

Fig. S4. Modelling force and torque distribution of different effectors.

Fig. S5. Magnetization profile to 2D magnetization curve and 3D magnetization mold.

Fig. S6. Validation of effector magnetization.

Fig. S7. Velocity quantification of differently magnetized deflecting effectors.

Fig. S8. Simulation analysis of radial linear motion magnetic soft machines.

Fig. S9. Independent and collaborative actuation of adjacently positioned linear motion magnetic soft machines.

Table S1. Comparison of different types of muscle actuators.

Legends for movies S1 to S7.

Other Supplementary Material for this manuscript includes the following:

Movie S1 (.mp4 format). Demonstration of deflecting magnetic soft machines with different magnetization profiles for S- and C-shaped motion.

Movie S2 (.mp4 format). Demonstration of lifting magnetic soft machines.

Movie S3 (.mp4 format). Demonstration of tilting magnetic soft machines.

Movie S4 (.mp4 format). Demonstration of petal-based magnetic soft machines.

Movie S5 (.mp4 format). Demonstration of radial linear motion magnetic soft machines.

Movie S6 (.mp4 format). Demonstration of deflecting magnetic soft machines for tilting of an interconnecting structure and human interaction.

Movie S7 (.mp4 format). Demonstration of grasping magnetic soft machines at the tip of a flexible probe.

Text

Supplementary Material 1 - Magnetization of Magnetic Soft Elements

Magnetic soft elements constitute elementary building blocks of effectors. They carry a one-dimensional non-uniform magnetization profile $(\boldsymbol{m}(y))$ along their longitudinal axis (y). Along their length, the elements experience a force $\boldsymbol{F}(y)$ and torque $\boldsymbol{\tau}(y)$ due to the coil near-field $(\boldsymbol{B}(y))$, shown in Supplementary Figure S4.1. Fixating the soft element results in a bending moment due to both force and torque. Desired element magnetization profile $(\boldsymbol{m}(y))$ is computed based on cost-functions that optimize total bending moment, force, or torque. These cost-functions are used to compute $\boldsymbol{m}(y)$ that allows lifting, center deflection, and linear motion (Fig. S4.2).

Lifting elements are fixated on the radial edges of the coil (Fig. S4.2). For notation simplicity we define $y \in [0, 2R]$, where $R = 6$ mm is the radius of the coil. The magnetization profile optimizes vertical forces within the central region above the coil 4 mm (4 mm $\leq y \leq 8$ mm), where axial gradients are strongest. At the coil extremities the magnetization profile optimizes bending moment for element deflection in the lifting direction. The associated cost function for lifting elements is thereafter defined as:

$$C_{\text{lift}}(\boldsymbol{m}(y)) = \left\{ \begin{array}{ll} \arg\max_{\boldsymbol{m}(y)} \hat{\boldsymbol{x}}^T \int_0^{y_1} \boldsymbol{m}(y) \times \boldsymbol{B}(y) + \langle 0, y, 0 \rangle \times \nabla(\boldsymbol{m}(y) \cdot \boldsymbol{B}(y)) \, dy, & y_1 = 4 \text{ mm} \\ \arg\max_{\boldsymbol{m}(y)} \hat{\boldsymbol{z}}^T \int_{y_1}^{y_2} \nabla(\boldsymbol{m}(y) \cdot \boldsymbol{B}(y)) \, dy, & y_2 = 8 \text{ mm} \\ \arg\min_{\boldsymbol{m}(y)} \hat{\boldsymbol{x}}^T \int_{y_2}^{y_3} \boldsymbol{m}(y) \times \boldsymbol{B}(y) + \langle 0, y - y_3, 0 \rangle \times \nabla(\boldsymbol{m}(y) \cdot \boldsymbol{B}(y)) \, dy, & y_3 = 12 \text{ mm}, \end{array} \right\} \tag{1}$$

where $\langle \cdot \rangle$ represents a column vector, $\hat{\boldsymbol{x}} = \langle 1, 0, 0 \rangle$, $\hat{\boldsymbol{z}} = \langle 0, 0, 1 \rangle$, and $\boldsymbol{m}(y) = \langle 0, m_y(y), m_z(y) \rangle$ computed numerically at discrete locations and given constant magnitude.

Similarly, center deflection and linear motion elements maximize bending moments relative

to the coil center, and linear forces planar to the coil surface:

$$C_{\text{defl}}(\boldsymbol{m}(y)) = \underset{\boldsymbol{m}(y)}{\arg\max}\, \hat{\boldsymbol{x}}^T \int_0^{y_3} \boldsymbol{m}(y) \times \boldsymbol{B}(y) + \langle 0, y - R, 0 \rangle \times \nabla(\boldsymbol{m}(y) \cdot \boldsymbol{B}(y))\, dy \tag{2}$$

$$C_{\text{lin}}(\boldsymbol{m}(y)) = \underset{\boldsymbol{m}(y)}{\arg\max}\, \hat{\boldsymbol{y}}^T \int_0^{y_3} \nabla(\boldsymbol{m}(y) \cdot \boldsymbol{B}(y))\, dy, \tag{3}$$

where $\langle 0, y - R, 0 \rangle$ is the moment arm with respect to the coil center (since $y \in [0, 2R]$).

Supplementary Material 2 - Validating Effector Magnetization

Magnetic soft effectors are assembled from one-dimensional pre-magnetized elements. The magnetization profile of the elements is reconstructed in terms of a magnetization angle $(\theta(y))$ at discrete locations along and with respect to their longitudinal axis (y). The discretized sub-volumes are considered point dipoles.

The soft element is placed on a non-magnetic acrylic table (Fig. S6). A tri-axis Hall-effect sensor (MLX 90371, Melexis, Ypres, Belgium) is moved above the element by a Panda robotic arm (Franka Emika, Munich, Germany). The Hall-effect sensor is connected to an Arduino Due microcontroller transmitting field measurements to an external PC at 100 Hz. Field measurements are taken along the longitudinal direction of the element $(y \in [y_{\min}, y_{\max}])$ at different heights $(z \in \{1, 2, ..., 10\}$ mm$)$. Sensor recording positions $(\boldsymbol{p}_{\text{r}} = \langle 0, y, z \rangle)$ and corresponding field measurements $(\boldsymbol{B}_{\text{r}}(\boldsymbol{p}_r) \in \mathbb{R}^3)$ are stored.

Reconstructed magnetization profiles of elements are defined by their unit dipole moment vectors $(\boldsymbol{m}_{\text{r}}(y))$. This profile is computed using a distributed point dipole model. We assume that each field measurement $(\boldsymbol{B}_{\text{r}}(\boldsymbol{p}_{\text{r}}))$ is the sum of the field by each distributed point dipole:

$$\boldsymbol{B}_r(\boldsymbol{p}_r) = \int_{y_{\min}}^{y_{\max}} \boldsymbol{\beta}(\boldsymbol{p}_{\text{r}}, y) \hat{\boldsymbol{m}}_{\text{r}}(y)\, dy, \tag{4}$$

where $\boldsymbol{\beta}(\boldsymbol{p}_\mathrm{r}, y) \in \mathbb{R}^{3\times3}$ represents the external magnetic field magnitudes produced by a magnetic point dipole irrespective of its direction, and $\hat{\boldsymbol{m}}_\mathrm{r}(y)$ represents the direction of the point dipole:

$$\boldsymbol{\beta}(\boldsymbol{p}_\mathrm{r}, y) = \frac{\mu_0\mu_c}{4\pi\|\Delta\boldsymbol{p}\|^5}\left(3\Delta\boldsymbol{p}\Delta\boldsymbol{p}^T - \Delta\boldsymbol{p}^T\Delta\boldsymbol{p}\,\boldsymbol{I}_{3\times3}\right), \tag{5}$$

$$\Delta\boldsymbol{p} = \boldsymbol{p}_r - \langle 0, y, 0\rangle, \tag{6}$$

with μ_0 the vacuum magnetic permeability, μ_c is the subvolume dipole moment magnitude, and $\boldsymbol{I}_{3\times3}$ the identity matrix.

The distributed point dipole model (Eqn. (4)) is discretized as $D \in \mathbb{Z}^+$ unit point dipoles $(\hat{\boldsymbol{m}}_\mathrm{r}^i,\ i \in \{1, 2, ..., D\})$ distributed uniformly between y_min and y_max. Thereafter, magnetization profile reconstruction from $M \in \mathbb{Z}^+$ field measurements $(\boldsymbol{B}_\mathrm{r}(\boldsymbol{p}_\mathrm{r}^j),\ j \in \{1, 2, ..., M\})$ becomes a linear least-squares problem,

$$\begin{bmatrix}\boldsymbol{B}_r(\boldsymbol{p}_r^1)\\ \vdots \\ \boldsymbol{B}_r(\boldsymbol{p}_r^M)\end{bmatrix} = \begin{bmatrix}\boldsymbol{\beta}(\boldsymbol{p}_r^1, y_\mathrm{min}) & \dots & \boldsymbol{\beta}(\boldsymbol{p}_r^1, y_\mathrm{max})\\ & \vdots & \\ \boldsymbol{\beta}(\boldsymbol{p}_r^M, y_\mathrm{min}) & \dots & \boldsymbol{\beta}(\boldsymbol{p}_r^M, y_\mathrm{max})\end{bmatrix}\begin{bmatrix}\hat{\boldsymbol{m}}_\mathrm{r}^1(y_\mathrm{min})\\ \vdots \\ \hat{\boldsymbol{m}}_\mathrm{r}^D(y_\mathrm{max})\end{bmatrix}. \tag{7}$$

For each sample we chose $D = 15$, $M \approx 25000$, y_min =-7.5 mm, and $y_\mathrm{max} = 7.5$ mm. The magnetization error of each element is quantified in terms of the reconstructed and desired angle $(\theta_\mathrm{r}(y), \theta_\mathrm{d}(y) \in \mathbb{S})$ between the longitudinal axis of the element (y) and the respective reconstructed and desired magnetization profile $(\boldsymbol{m}_\mathrm{r}(y), \boldsymbol{m}(y))$. The average angular error across all samples is 14.74^o.

Supplementary Figures

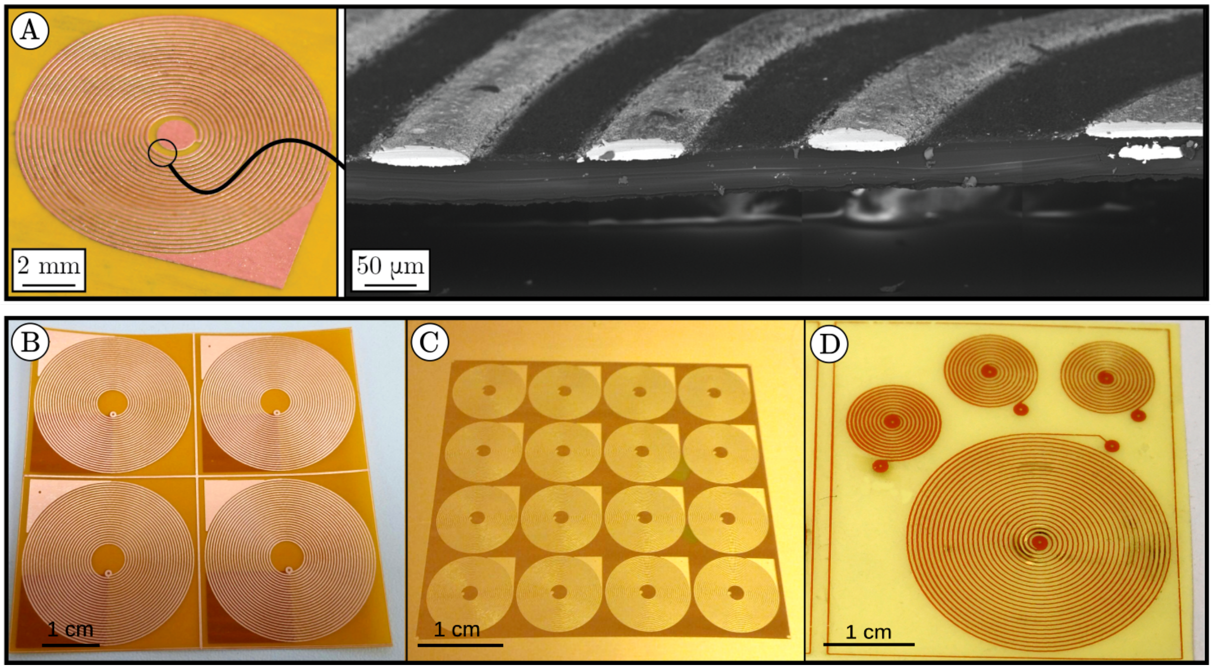

Fig. S1: Planar electromagnetic coil geometries. **(A)** Spiral planar copper coil with 24 revolutions used in this work. The coil has an inner and outer diameter of 2 mm and 12 mm, respectively. A copper coating with vertical thickness of 9 μm is attached to a polyimide laminate substrate of thickness 50 μm. Horizontal thickness and spacing of wires is 100 μm. **(B)** Coil diameters can be made arbitrarily bigger or **(C)** smaller, and prepared as individual units or as arrays of coils in a grid-like pattern on the same laminate substrate. **(D)** The symmetry of the array of coils can be freely chosen. Additionally, the array can consist of coils of different dimensions.

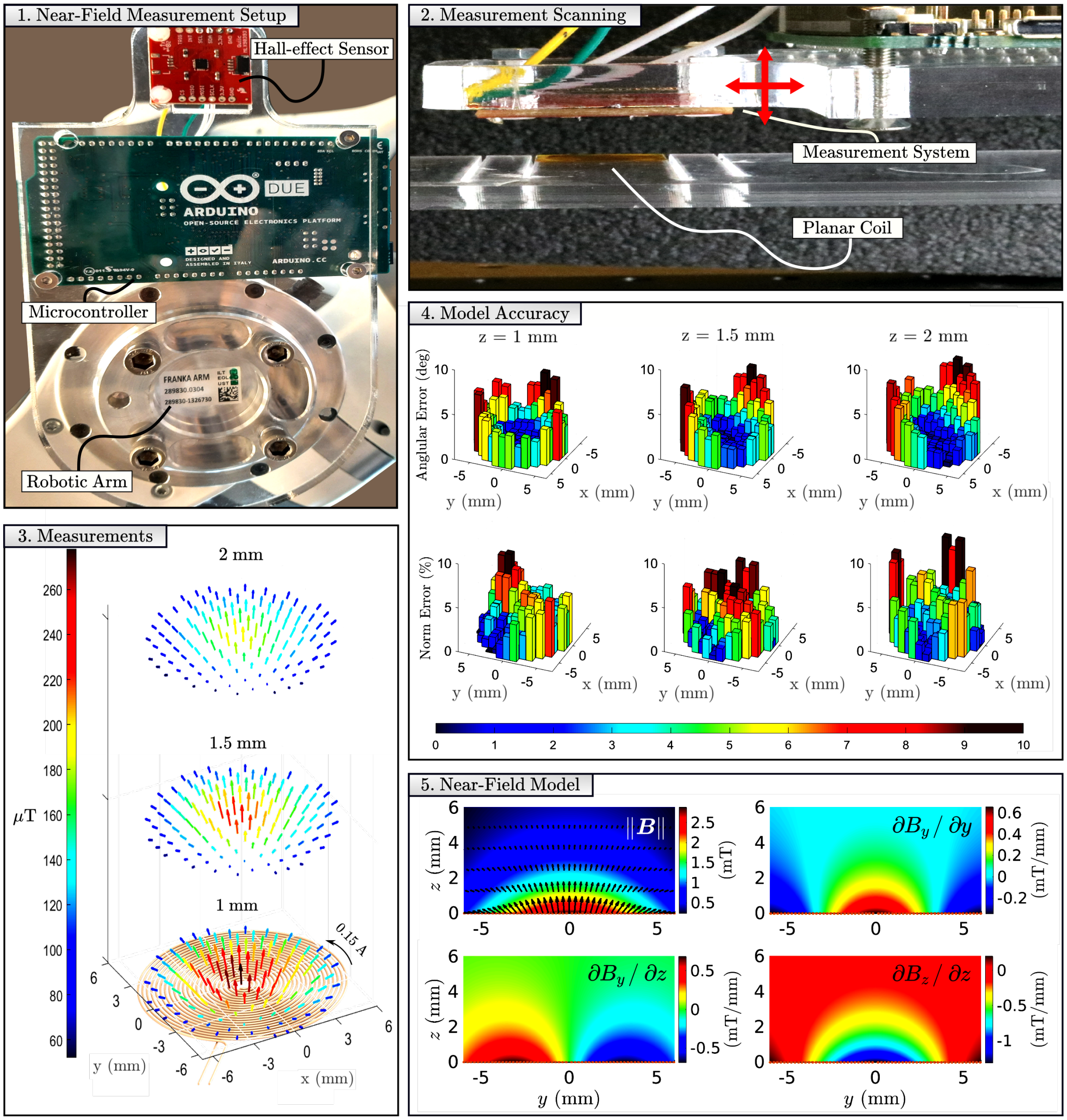

Fig. S2: Measurement and model results for magnetic near-field. **(1)** Measurement setup comprising a tri-axis Hall-effect sensor, Arduino Due microcontroller, and Franka Emika Panda robotic arm. **(2)** Field measurements are performed with a volumetric sweep above the coil surface at distances 1, 1.5, and 2 mm. **(3)** Measurements obtained at coil operating current of $I = 0.15$ A. The shown data are compensated for earth magnetic field. **(4)** Model accuracy. Angular discrepancy between measured and modeled field direction is within 1-3 ° in the central region of the coil and increases to 4-7 ° towards the edge. Norm discrepancy across the coil surface is in the range of 3-8%. **(5)** Modeled field magnitudes ($\|\boldsymbol{B}\| = \|\langle B_x, B_y, B_z\rangle\|$) and non-zero gradients in the yz-plane above the coil for a current $I = 1$ A.

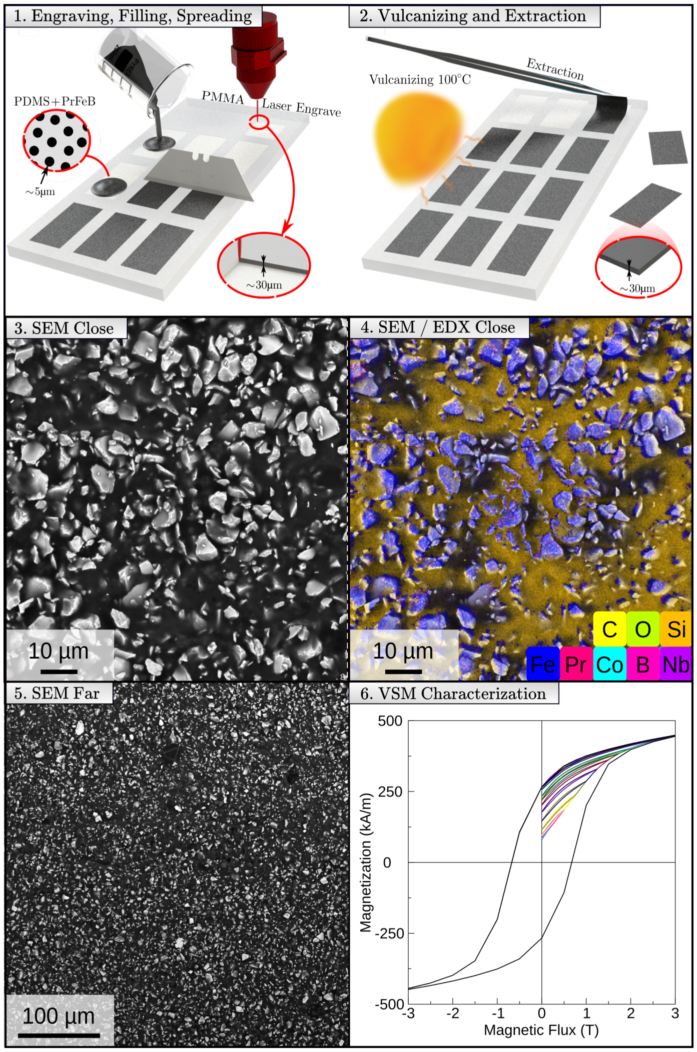

Fig. S3: Fabrication and characterization of magnetic soft polymer composite (MPC) sheets. (1) Rectangular cavities of depth 30 μm are laser-engraved (Speedy 300, Trotec Laser, Marchtrenk, Austria) in an acrylic/PMMA plate. A suspension of polydimethylsiloxane (PDMS) and ferromagnetic microparticles (Pr-Fe-Co-Nb-B, MQP-16-7-11277-070, Magnequench GmbH, Germany) with mean diameter of 5 μm and volume fraction 0.35 is poured inside the cavities. The suspension is uniformly distributed in the molds and excess removed by sliding a Stanley knife. **(2)** Vulcanization of the suspension is performed at 100 °C for 1 h. The resulting magnetic soft composite sheets are thereafter extracted from the cavities. **(3)** Scanning electron microscopy (SEM) image of the top surface of a sheet. **(4)** EDX element analysis of the corresponding area. The element analysis identifies the chemical composition of the magnetic microparticles within the PDMS matrix. **(5)** Large field of view SEM image of the sheet. Magnetic microparticles are evenly distributed within the polymer matrix. **(6)** SQUID-VSM magnetic characterization of a uniformly magnetized sheet. Minor hysteresis loops reveal robustness of the predefined magnetic state of the sheets. The sheet retains its remanent magnetization of 25 kA/m even upon application of a magnetic field up to 0.5 T. The sheets have a coercivity of 0.7 T.

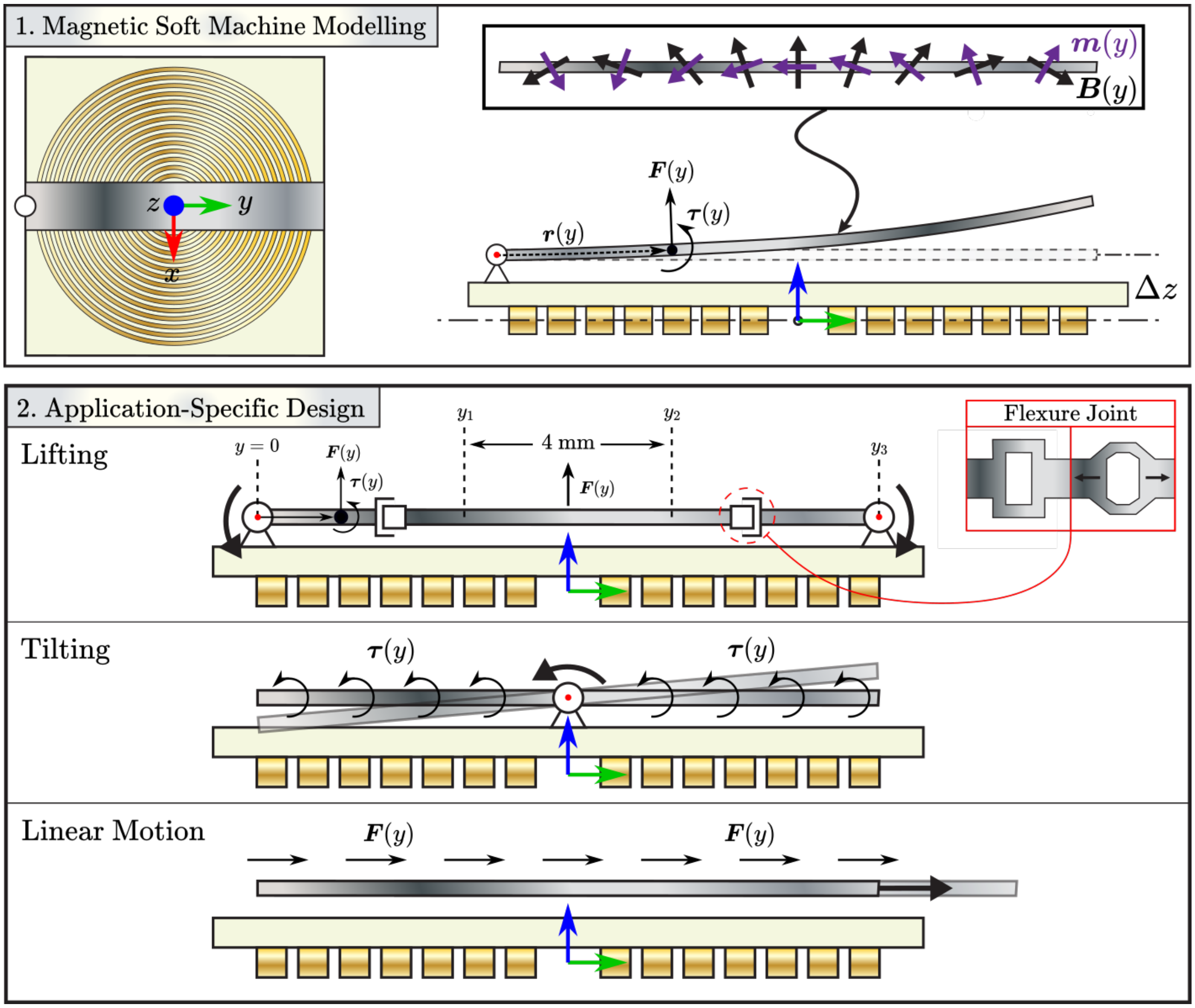

Fig. S4: Modelling principles of magnetic soft machines (MSMs) as assemblies of magnetized soft elements. **(1)** Soft elements are considered one-dimensional strips on a coil, at non-zero height (Δz), with a magnetization profile ($\boldsymbol{m}(y)$) along their longitudinal axis (y). Interaction between $\boldsymbol{m}(y)$ and coil near-field ($\boldsymbol{B}(y)$) results in distributed forces ($\boldsymbol{F}(y) = \nabla(\boldsymbol{B}(y) \cdot \boldsymbol{m}(y))$) and torques ($\boldsymbol{\tau}(y) = \boldsymbol{m}(y) \times \boldsymbol{B}(y) + \boldsymbol{r}(y) \times \boldsymbol{F}(y)$), where $\boldsymbol{r}(y)$ represents a moment arm. **(2)** Application-specific magnetic force and torque distributions for lifting, tilting, and linear motion MSMs. Lifting elements are split in three sections: two extremities $\{(y_0\text{-}y_1), (y_2\text{-}y_3)\}$ and one center $(y_1\text{-}y_2)$. The left and right extremities are optimized for counter- and clockwise bending moments as a result of both forces and torques. The center is optimized for vertical forces. Flexure joints within the extremities allow extension during vertical displacement of the lifting element. Tilting elements are optimized for bending moments with respect to the central axis. Linear motion elements are optimized for planar forces.

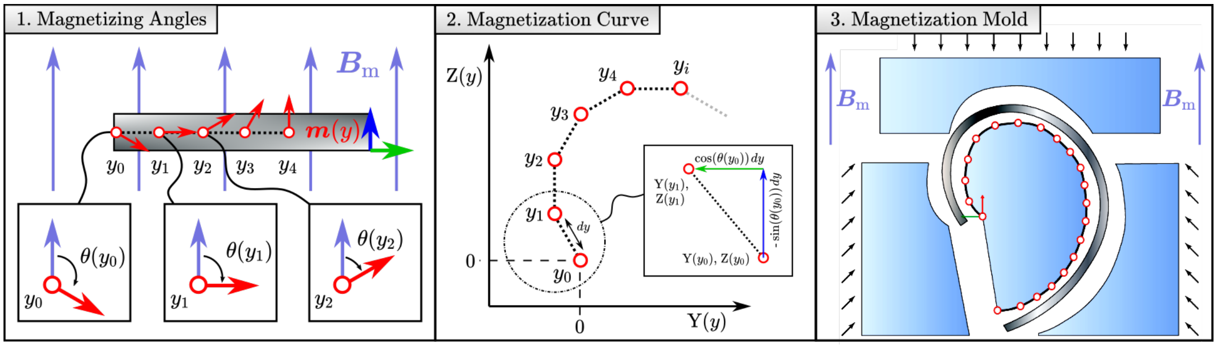

Fig. S5: Computing magnetization curve and associated mold for a desired magnetic soft composite sheet magnetization profile $(\boldsymbol{m}(y))$ in an external homogeneous field $(\boldsymbol{B}_{\mathrm{m}})$. (1) Angular offset between desired magnetization profile and magnetizing field $(\theta(y) = \angle \boldsymbol{m}(y), \boldsymbol{B}_{\mathrm{m}})$ is computed along the longitudinal axis of the sheet (y). **(2)** A magnetization curve $(\mathrm{Y}(y), \mathrm{Z}(y))$ is computed using forward Euler integration: $\mathrm{Y}(y) = \int_0^y \cos(\theta(\sigma))d\sigma$ and $\mathrm{Z}(y) = \int_0^y -\sin(\theta(\sigma))d\sigma$. The magnetization curve represents a physical wrapping of the sheet which produces the desired magnetization profile when subjected to the magnetizing field. **(3)** The 2D magnetization curve is extruded to form a 3D mold. Wrapping the sheet inside the mold and subjecting to a uniform magnetizing field $(\boldsymbol{B}_{\mathrm{m}})$ produces the desired magnetization profile.

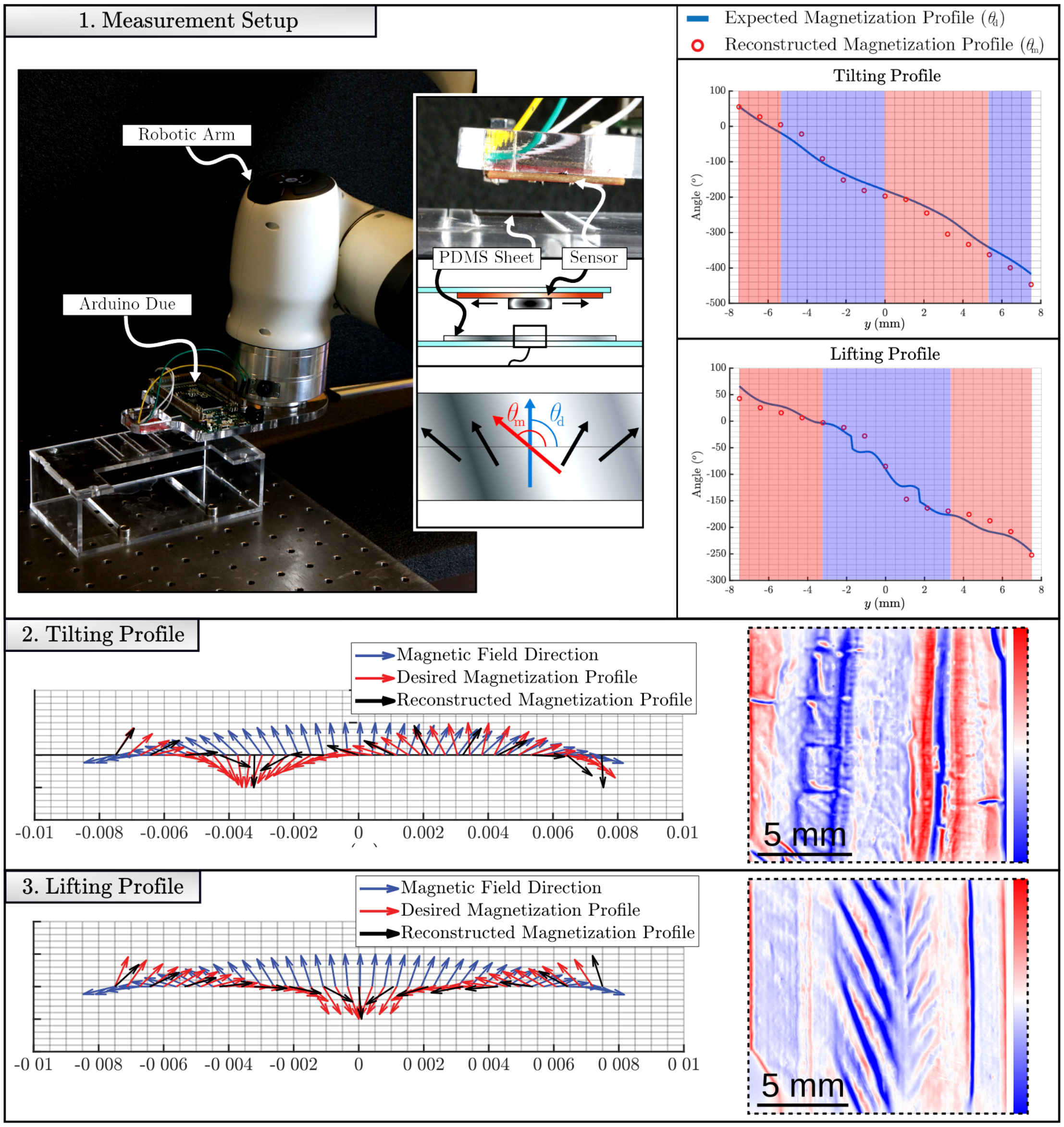

Fig. S6: Validating magnetization of magnetic soft effectors. A Tri-axis Hall-effect sensor connected to an Arduino Due microcontroller and robotic arm is swept longitudinally over soft effectors. Magnetization direction is reconstructed in terms of a measured magnetization angle (θ_m) relative to the longitudinal element axis (y), and compared to the desired magnetization angle (θ_d). Validation is performed for soft effectors designed for tilting and lifting MSMs. Desired and reconstruction magnetization profiles are shown, as well as magnetic field directions on the planar coil surface. Stray fields measured directly on the element surface are shown, with red and blue colors indicating ”out-of-page” and ”into-page” magnetization.

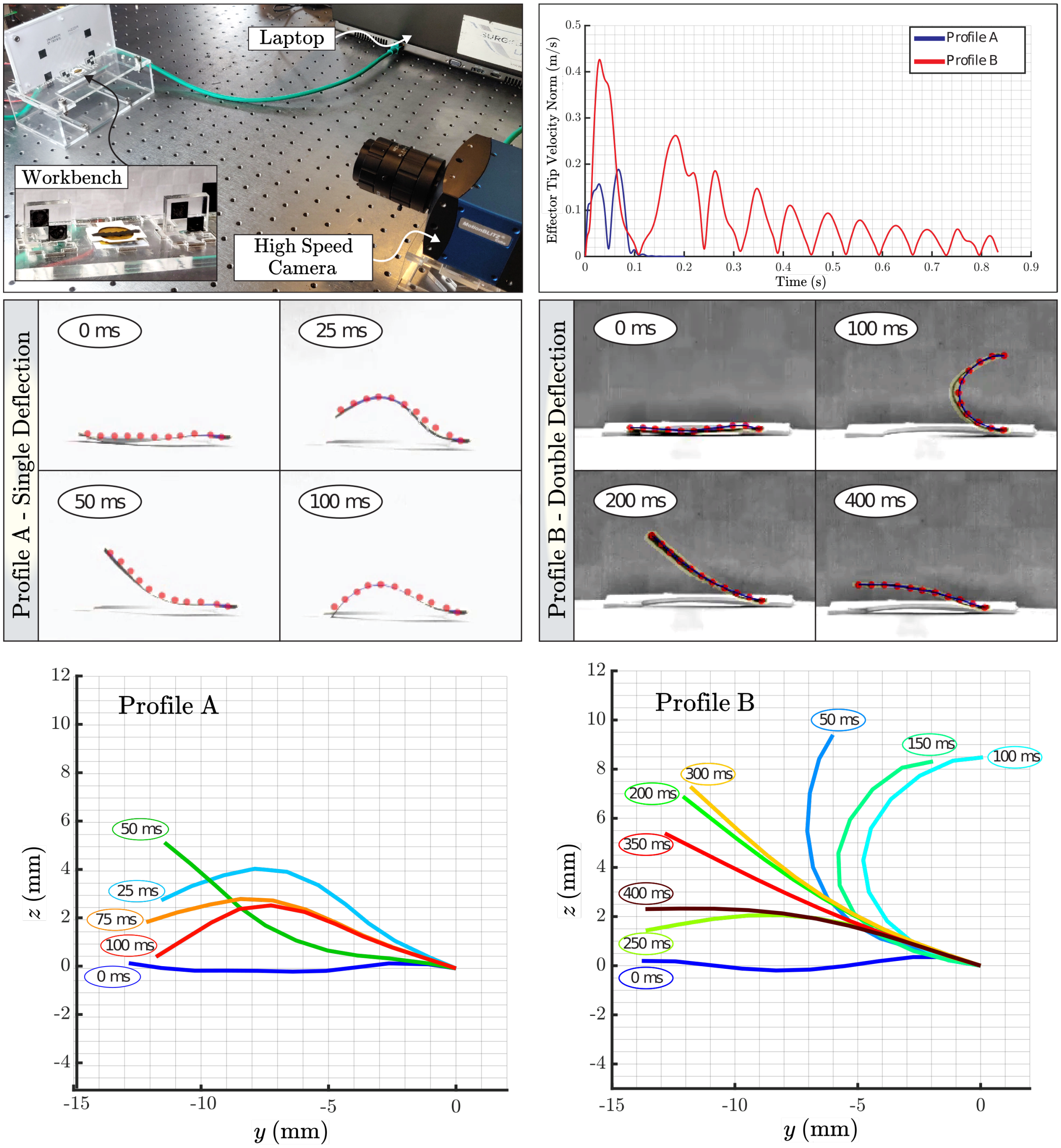

Fig. S7: Motion profile of magnetic soft effectors with different magnetization profiles (A and B) designed for single and double deflection. Coils are powered with a step current of 1 A. Effector motion is captured with a high speed camera, their longitudinal axis reconstructed, and tip velocity magnitude computed. Effector configurations are shown at different time constants.

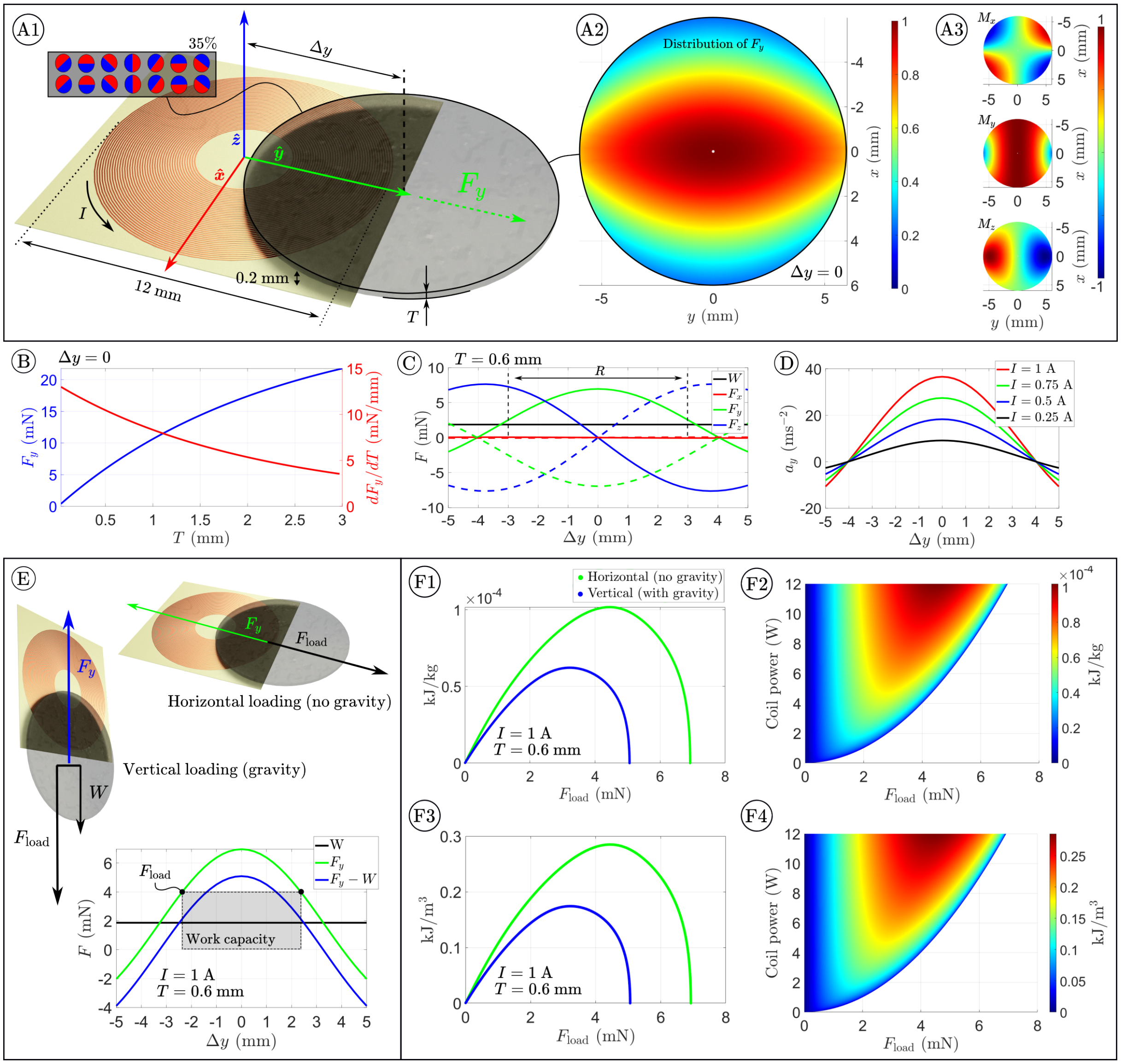

Fig. S8: Simulation analysis of radial linear actuators assuming no friction. **(A1)** Planar coil of diameter $(2R = 12\ \mathrm{mm})$ running current (I) with a disk-shaped effector of thickness (T) positioned $0.2\ \mathrm{mm}$ above the surface. The effector has a magnetic volume fraction of $35\ \%$ and experiences a radial force $(F_y(\Delta y))$ depending on displacement (Δy) with respect to the coil center. **(A2)** Distribution of F_y across the effector surface when concentric with the coil for **(A3)** force-optimized magnetization $(\boldsymbol{M} = \langle M_x, M_y, M_z \rangle)$ profiles. **(B)** Total F_y on the effector for varying thickness (T) at $\Delta y = 0$ and $I = 1$ A. **(C)** Weight (W) and magnetic forces on an effector of thickness $T = 0.6\ \mathrm{mm}$ for displacements (Δy) at currents $I =$-1 A (dashed) and $I = 1$ A (solid), with **(D)** effector acceleration for different I. **(E)** Performable work by the effector on a load (F_{load}) with and without gravity. **(F1)** Work capacity of the effector for varying F_{load} at **(F2)** different coil power consumption (resistance $12\ \Omega$). **(F3)** Energy density of the effector for varying F_{load} at **(F4)** different coil power consumption.

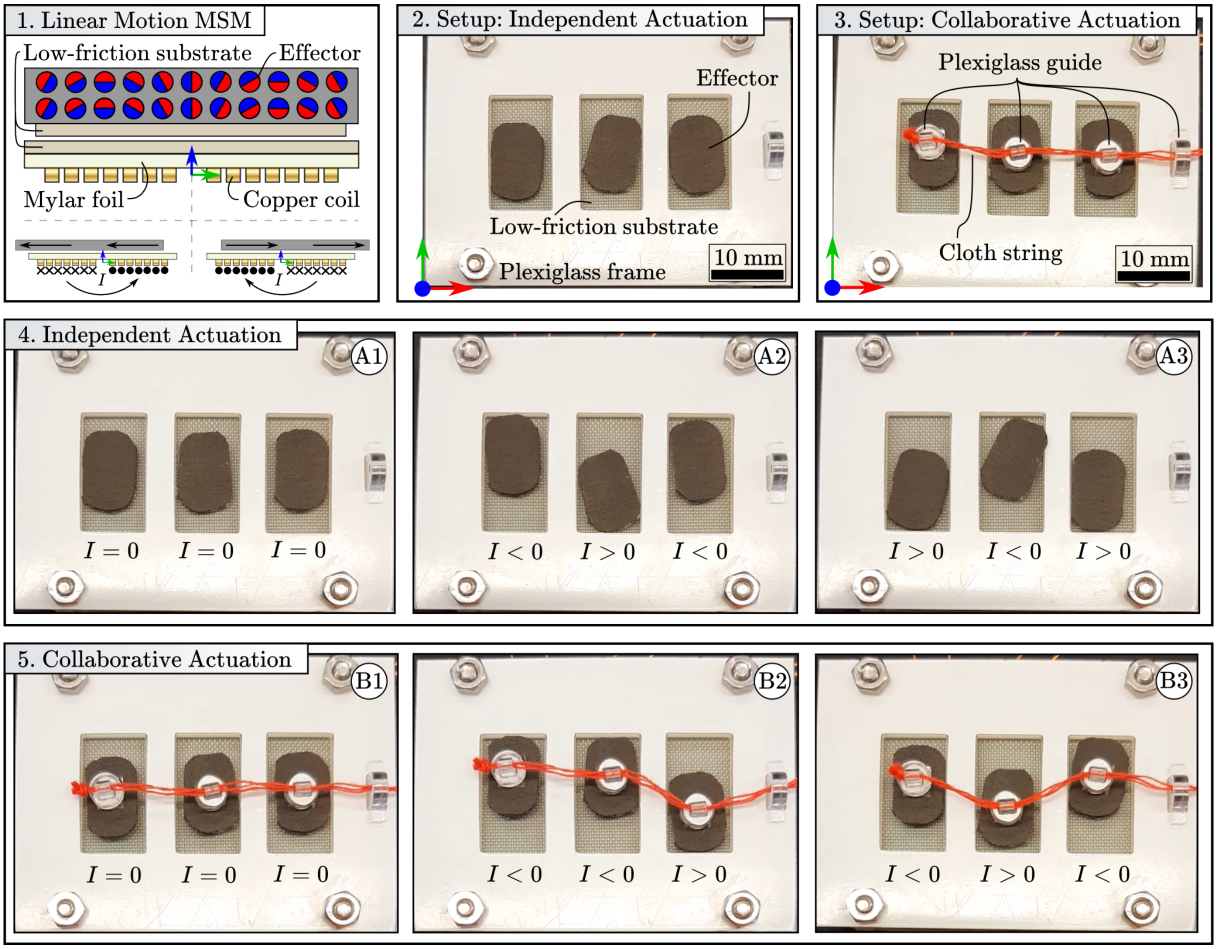

Fig. S9: Independent and collaborative actuation of adjacently positioned linear motion magnetic soft machines (MSMs). **(1)** Schematic representation of the linear motion MSM. A copper coil (thickness $9\ \mu\text{m}$) is attached to a mylar polyimide foil ($41\ \mu\text{m}$), which is covered by a low-friction substrate comprising PTFE-coated fiberglass ($100\ \mu\text{m}$). The magnetoresponsive effector ($600\ \mu\text{m}$) has radial force-optimized magnetization profile and is also covered below by the low-friction substrate. The coil plane (yz-plane) is marked by the green and blue arrow. Depending on the direction of current (I) through the coil, the effector displaces along the positive or negative y-axis. **(2)** Setup to demonstrate independent actuation of three linear motion MSMs. Coils are not visible, but are positioned central below each rectangular channel. **(3)** Setup to demonstrate collaborative actuation of three linear motion MSMs. A cloth string is attached to each effector. **(4)** Demonstration of independent actuation. From the neutral configuration (A1), impulse currents ($I = 0.6$ A) are applied (A2) and subsequently reversed (A3). **(5)** Demonstration of collaborative actuation. From the neutral configuration (B1), impulse currents ($I = 0.6$ A) are applied to actuate effectors to form an S-shape (B2-B3). Also see movie S5.

Type	Size (mm)	Strain (%)	Work capacity (kJ/kg)	Work density (kJ/m^3)	Bandwidth (Hz)	Ref.
HSM	-	30	25	25	10	[80]
HFA	$\varnothing 3.2 \times 130$	115.7	0.15	-	25	[81]
TPA	$\varnothing 1 \times 110$	30.6	3.5	2000	0.4	[44]
MKA	$\varnothing 31 \times 110$	38	1.5	0.3	10	[82]
CAM	$\varnothing 4.6 \times 100$	50	0.38	-	4	[83]
HAS	$\varnothing 50 \times 15$	60	0.07	0.23	20	[84]
**Type**	**Size** (mm)	**Stroke** (%)	**Work capacity** ($kJ/(kg \cdot A)$)	**Work density** ($kJ/(m^3 \cdot A)$)	**Bandwidth** (Hz)	**Ref.**
MNF	$\varnothing 10 \times 10$	25	$6 \cdot 10^{-6}$	0.056	40	[64]
MNF	$\varnothing 9 \times 4.7$	25	-	$0.25 \cdot 10^{-3}$	45	[65]
MNF	$\varnothing 12 \times 0.08$	2000	$1.2 \cdot 10^{-4}$	0.29	25	This work (lifting)
MNF	$\varnothing 12 \times 0.85$	± 25	$1 \cdot 10^{-4}$	0.3	25	This work (radial motion)

Table S1: Comparison of different types of muscle actuators in terms of size, actuation strain or stroke, work capacity, work density, and bandwidth. HSM, human skeletal muscle; HFA, hydraulic filament actuator; TPA, twisted polymer actuator; CAM, cavatappi artificial muscle; HAS, HASEL: hydraulically amplied self-healing electrostatic; MNF, magnetic near-field. Note that for magnetic actuators stroke is reported instead of strain, because the working principle does not involve contraction or relaxation. Strain and stroke is defined with respect to the associated actuator dimension. Also, work capacity and work density are normalized with respect to driving currents. Although our work reports near-field MSM operation at frequencies up to 25 Hz, this is not the maximum achievable bandwidth. Listed values are approximations where the indication "$\sim$" has been omitted for compactness.

References

[44] Mingtong Li, Yichao Tang, Ren Hao Soon, Bin Dong, Wenqi Hu, Metin Sitti, *Sci. Adv.* **2022**, *8* (10), eabm5616.

[64] William R Johnson, Stephanie J Woodman, Rebecca Kramer-Bottiglio, *IEEE Int. Conf. Soft Robot.* **2022**, 761–766.

[65] Thanh Nho Do, Hung Phan, Thuc-Quyen Nguyen, Yon Visell, *Adv. Funct. Mater.* **2018**, *28* (18), 1800244.

[80] Meng Li, Aniket Pal, Amirreza Aghakhani, Abdon Pena-Francesch, Metin Sitti, *Nat. Rev. Mater.* **2022**, *7* (3), 235–249.

[81] Phuoc Thien Phan, Trung Thien Hoang, Mai Thanh Thai, Harrison Low, Nigel Hamilton Lovell, Thanh Nho Do, *Soft Robot.* **2022**, *9* (4), 820–836.

[82] Daniel Villegas, Michaël Van Damme, Bram Vanderborght, Pieter Beyl, Dirk Lefeber, *Adv. Robot.* **2012**, *26* (11-12), 1205–1227.

[83] Diego R Higueras-Ruiz, Michael W Shafer, Heidi P Feigenbaum, *Sci. Robot.* **2021**, *6* (53), eabd5383.

[84] Eric Acome, Shane K Mitchell, TG Morrissey, MB Emmett, Claire Benjamin, Madeline King, Miles Radakovitz, Christoph Keplinger, *Science* **2018**, *359* (6371), 61–65.

Supporting Movies

Movie S1. Demonstration of magnetic soft machines (MSMs) with different effector magnetization profiles for double- and single-deflection motion.

Movie S2. Demonstration of a magnetic soft machine (MSM) with an effector magnetization profile designed for lifting motion in the coil magnetic near-field. The video is divided in four clips. 1) Lifting MSM moving vertically without a payload. 2) Lifting MSM lifts and launches a $3 \times 3 \times 3\ \mathrm{mm}^3$ cube of weight $27\ \mathrm{mg}$. 3) Lifting MSM rapidly vibrates and shakes off a $3 \times 3 \times 3\ \mathrm{mm}^3$ cube of weight $27\ \mathrm{mg}$ from itself. 4) Lifting MSM attempts to lift a $4 \times 4 \times 4\ \mathrm{mm}^3$ cube of weight $64\ \mathrm{mg}$ resulting in tilting motion.

Movie S3. Demonstration of a magnetic soft machine (MSM) with an effector magnetization profile designed for rotational tilting motion about the central axis. The video is divided in three clips. 1) Tilting MSM without a payload. 2) MSM tilts a $3 \times 3 \times 3\ \mathrm{mm}^3$ cube of weight $27\ \mathrm{mg}$. 3) MSM tilts a $4 \times 4 \times 4\ \mathrm{mm}^3$ cube of weight $64\ \mathrm{mg}$.

Movie S4. Demonstration of a magnetic soft machine (MSM) with an effector composed of several petal-shaped elements with magnetization profiles designed for bending moments relative to the center of the coil. The video is divided in five clips. 1) MSM with two petals of opposing magnetization deflect in phase shifting currents through the coil. 2) High-speed recording of an MSM with two petals moving at $25\ \mathrm{Hz}$. 3) Adjacent MSMs with similar and dissimilar petals are activated independently. 4) MSM with similarly magnetized petals shows gripper-like motion. 5) Adjacent MSMs are activated independently underwater.

Movie S5. Demonstration of magnetic soft machines (MSMs) with effector magnetization profiles designed for planar linear motion, acting as a soft muscle. The video is divided in five clips. 1) Linear motion MSM showing horizontal movement within a flexible holder on a film of water to reduce friction. 2) Linear motion MSM that is manually pulled and exhibits spring-like behavior. 3) Linear motion MSM on a low-friction substrate showing horizontal movement without additional lubrication. 4) Linear motion MSM on a low-friction substrate moving an M4 aluminium nut of weight $650\ \mathrm{mg}$. 5) Interconnected linear motion MSMs showing collaborative pushing and pulling. 6) Adjacent linear motion MSMs with bidirectional motion on single coils. 7) Adjacent linear motion MSMs with collaborative action.

Movie S6. Demonstration of magnetic soft machines (MSMs) with effector magnetization profiles designed for bending moments relative to a rigid attachment point on the radial edge of the coil. The video is divided in four clips. 1) Deflecting effector with a magnetization profile optimized for bending moments, showing S-shaped deflection after activation. 2) Interconnecting effectors to tilt the connecting structure. 3) Effector of circular geometry with a torque-constrained magnetization profile, with magnetic torques strictly in direction of deflection. 4) Adjacent effectors of circular geometry activated independently with human interaction for, e.g., gaming, rehabilitation, and concentration practice.

Movie S7. Demonstration of assembly of magnetic soft machines (MSMs) as functionalized elements on a flexible probe tip. The video is divided in three clips. 1) Bending of the flexible tip underwater showing deformation-independent functionality of MSMs. 2) Adjacent positioning of MSMs activated independently. 3) MSMs grasping and releasing a payload of weight $20\ \mathrm{mg}$.
